# Supplementary material for: Correction: An integrated approach for advanced vehicle classification
Source: PLoS One. 2025 Jun 10;20(6):e0326073. doi: 10.1371/journal.pone.0326073 (PMC12151334; doi:10.1371/journal.pone.0326073)
Supplement: S2 File — (PDF) [file pone.0326073.s002.pdf]

RESEARCH ARTICLE

An integrated approach for advanced vehicle classification

Rui Liu☯*, **Shiyuan Wen**☯, **Yufei Xing**☯

College of Computer Science and Cyber Security, Chengdu University of Technology, Chengdu, Sichuan, China

☯ These authors contributed equally to this work.
* liu.rui2@student.zy.cdut.edu.cn

Check for updates

OPEN ACCESS

Citation: Liu R, Wen S, Xing Y (2025) An integrated approach for advanced vehicle classification. PLoS One 20(2): e0318530. https://doi.org/10.1371/journal.pone.0318530

Editor: Xiyu Liu, Shandong Normal University, CHINA

Received: July 14, 2024

Accepted: January 16, 2025

Published: February 18, 2025

Copyright: © 2025 Liu et al. This is an open access article distributed under the terms of the Creative Commons Attribution License, which permits unrestricted use, distribution, and reproduction in any medium, provided the original author and source are credited.

Data availability statement: All data files are available from the database (http://datadryad.org/stash/share/RGbzSPJW0qAa4P5pdIexqE12xssvdNjYJxhF60YT6QY).

Funding: The author(s) received no specific funding for this work.

Competing interests: The authors have declared that no competing interests exist.

Abstract

This study is dedicated to addressing the trade-off between receptive field size and computational efficiency in low-level vision. Conventional neural networks (CNNs) usually expand the receptive field by adding layers or inflation filtering, which often leads to high computational costs. Although expansion filtering was introduced to reduce the computational burden, the resulting receptive field is only a sparse sampling of the tessellated pattern in the input image due to the grid effect. To better trade-off between the size of the receptive field and the computational efficiency, a new multilevel discrete wavelet CNN model (DWAN) is proposed in this paper. The DWAN introduces a four-level discrete wavelet transform in the convolutional neural network architecture and combines it with Convolutional Block Attention Module (CBAM) to efficiently capture multiscale feature information. By reducing the size of the feature maps in the shrinkage subnetwork, DWAN achieves a wider sensory field coverage while maintaining a smaller computational cost, thus improving the performance and efficiency of visual tasks. In addition, this paper validates the DWAN model in an image classification task targeting fine categories of automobiles. Significant performance gains are observed by training and testing the DWAN architecture that includes CBAM. The DWAN model can identify and accurately classify subtle features and differences in automotive images, resulting in better classification results for the automotive fine-grained category. This validation result further demonstrates the effectiveness and robustness of the DWAN model in vision tasks and lays a solid foundation for its generalization to practical applications.

Introduction

Image processing plays an essential role in artificial intelligence, such as interpreting the surroundings in the public's daily life. For the past few decades, there have been more and more "urban diseases", taking the significant deterioration of the traffic environment because of the significant expansions of urban areas. Under these circumstances, surveillance systems based on computer-vision models are the most efficient technologies with limited human resources [1]. The common methods of vehicle traffic monitoring include the recognition of vehicle signs based on small targets, and the recognition based on the overall characteristics of vehicles [2]. However, the task of vehicle detection and classification is challenging. Traffic surveillance

systems need to process a large number of images and videos to extract the characteristics of various vehicles. Nevertheless, in most cases, the surveillance data does not illustrate a general view of the vehicle. In addition, in the case of using the existing traditional technology, the detection time is often long, and the accuracy in the complex environment cannot fully meet the usage requirements. This creates a big obstacle for deep learning models to identify vehicle information by extracting features from images [3]. Nowadays, the attention mechanism is the most advanced and popular technology in the field of deep learning, especially after the Bert model was proposed, which has lower complexity than the current neural networks, such as CNN, RNN [4], and so on. The advantage of the attention mechanism is that high performance and speed can be achieved with fewer parameters. The attention mechanism can be added to the CNN structure because there is no dependency between the results of two computation steps in the attention mechanism [5]. Besides, the data needs to be pre-processed before building the proposed model, and the application of wavelet transform in the data pre-processing stage helps in separating the fine or rough parts of the image. Wavelet transform techniques can remove noise [6] and compress images [7] without causing any significant damage to the data. This paper aims to construct a depth-wise wavelet attention network that combines deep convolution neural networks (DCNN) [8] with an attention mechanism [9]. Firstly, this work will develop a depth-wise wavelet attention network framework for vehicle-type classification. After training and testing the network, other CNN models with different architectures such as VGG [10], AlexNet [11], ResNet [12], etc., will be compared with the depth-wise wavelet attention network based on various metrics, including accuracy, loss, recall, and F1-score, in the comparative analysis section. Both the concept of wavelet transforms [13] applied in the data preprocessing phase and the attention mechanism as the layer for CNNs aim to improve the results of feature extraction significantly. Compared to traditional CNN models, the depth-wise wavelet attention networks have higher performance in image classification tasks.

Many deep-learning models have been used for vehicle classification in recent years, most of which can be categorized into three types, including specifications mode, series mode, and brand mode [14].

Chen et al. [14] proposed a method for the first type. Their method only reserved three layers consisting of the convolution layer, the Max-pooling layer, and the fully connected layer with the SoftMax classifier. Based on the small-scale dataset they built, their model achieved a vehicle-type classification accuracy of 97.88% on the test set. However, such a high performance is based on a small sample size and few vehicle types. Furthermore, Zhao et al. [15] designed two architectures called CNNVA-Rule and CNNVA-RL, which consist of CNN with a visual attention mechanism for image classification. These architectures used a visual attention-based module to highlight one part of the image and weaken other parts to generate focused images. In experiments, both structures were consistently tested with over 96.00% accuracy in two different classes. However, the method they used to select viewpoints based on hash functions and manual rules was too subjective and costly. Additionally, attention mechanisms have become popular in recent years, Nasaruddin et al. [16] proved the positive impact of the attention mechanism on their proposed model, with a 14.30% increase in accuracy after adding the attention module.

Among the current methods for classifying vehicle types appearing in traffic surveillance images, it is common to distinguish small cars and large trucks by vehicle appearance features, but it is difficult to distinguish sedans, SUVs, and MPVs that have similar appearances. In addition, images obtained from road surveillance are noisy and only capture the local appearances of vehicles. Therefore, most of the recent studies use deep learning methods rather than pattern matching or image segmentation based on vehicle feature information to classify vehicle types in surveillance images.

The method proposed by Awang et al. [17] utilized an enhanced sparse-filtered convolutional neural network with a layer-skipping strategy (TC-SF-CNNLS) for vehicle-type classification. The technique extracts local and global features from the luminance and chromatic components of vehicle images, inspired by the human visual system's sensitivity to color and brightness. The TC-SF-CNNLS has been tested with a benchmark dataset and a self-obtained dataset, demonstrating high accuracy, precision, recall, and F-score in classifying vehicles into various classes, including those with similar features. Guo et al. [18] presented a semisupervised vehicle-type classification scheme using an ensemble of broad learning system (BLS) classifiers. This scheme is designed to overcome the limitations of traditional supervised learning in intelligent transportation systems (ITS) by leveraging both labeled and unlabeled data. The method includes training a collection of base BLS classifiers using semisupervised learning and constructing a dynamic ensemble structure for superior generalization performance. Experiments on public datasets have shown that this method outperforms single BLS classifiers and other mainstream methods in effectiveness and efficiency. Zhao et al. [19] introduced an optimized YOLOv4 model named YOLOv4-AF for vehicle detection and classification. This model incorporates an attention mechanism to suppress interference features in images and modifies the Feature Pyramid Network (FPN) part of the Path Aggregation Network (PAN) to enhance effective features through down-sampling. The YOLOv4-AF model has shown improved performance over the original YOLOv4 as well as other state-of-the-art models like Faster R-CNN and EfficientDet in terms of mean average precision (mAP) and F1-score on public datasets. Yu et al. [20], on the other hand, proposed a convolutional neural network (CNN) model with embedded vehicle pose information, known as the embedding pose CNN (EP-CNN), to address the multiview vehicle model recognition (MV-VMR) problem. The EP-CNN model includes a pose estimation subnetwork (PE-SubNet) for extracting vehicle pose information and a vehicle model classification subnetwork (VMC-SubNet) that integrates the pose features for classification. This approach has demonstrated higher recognition accuracy on benchmark datasets compared to several classic CNN models and state-of-the-art fine-grained vehicle model classification algorithms. A method based on the YOLO v3 model has been proposed by Park et al. [21], for recognizing three types of traffic vehicle images collected by UAVs at intersections, including passenger cars, trucks, and buses, which has the advantage of allowing fast classification of open roadways using aerial images, but is unable to accurately classify vehicles, especially those with similar features and lengths, such as passenger cars and lorries.

As shown in Table 1, the classification and application methods, vehicle types, and classification accuracy are summarized.

Materials and methods

In this paper, the proposed framework consists of three modules in Fig 1, including data input, representation, and classification.

Table 1. Comparison of vehicle classification.

Methods	Vehicle Types	Train Data Size	Accuracy (%)
SF-CNN, TC-SF-CNNLS, LSS [17]	Bus, SUV, Passenger car, Minivan, Truck, Taxi	2,700	90.50
BLS, SSL, ES [18]	Bus, Microbus, Minivan, Sedan, SUV, Truck	4,300	94.63
YOLOv4_AF, CBAM, FPN [19]	Bus, Microbus, Minivan, SUV, Sedan, Truck, Van	19,200	89.50
EP-CNN, Multi-SEBlock, HAM [20]	CompCars Dataset (431 types), Stanford Cars dataset (196 types)	43,560	93.50
YOLOv3 [21]	Sedan, Truck, Bus	3,279	95.10

https://doi.org/10.1371/journal.pone.0318530.t001

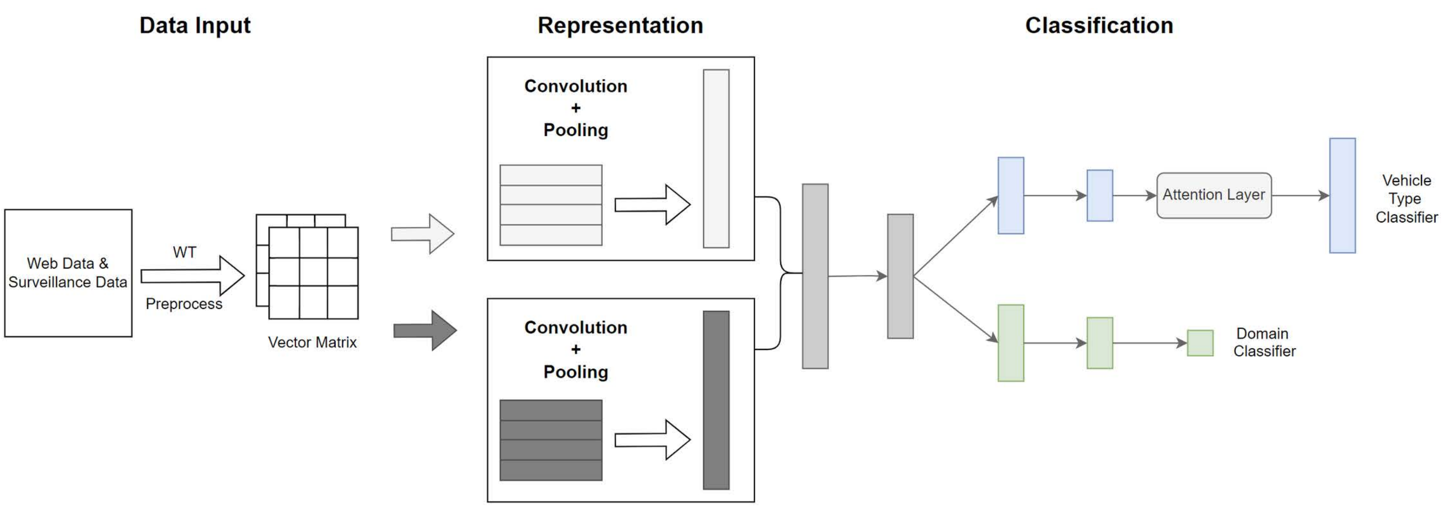

Fig 1. Architecture for vehicle type classification based on deep wavelet attention network.

https://doi.org/10.1371/journal.pone.0318530.g001

Data input

The source dataset (both web data and surveillance data) needs to be pre-processed, including resizing, image edge extraction, contrast-limited adaptive histogram equalization, image augmentation, and so on. Additionally, the wavelet transform technique will be applied to denoise the image.

Wavelet transform (WT). Compared with Fourier and Gaber transforms, it has the advantages of multi-resolution analysis and time-frequency localization, and can adaptively adjust the size of the window according to the frequency, so as to accurately capture the global features and local details of the signal at different scales. Generally, wavelet transform is divided into continuous and discrete [22]. In this paper, the discrete wavelet transform will be used to preprocess the data with the following formula:

$$W_{\varphi}(a,b) = \frac{1}{\sqrt{a}} \sum_{n} \varphi * \frac{(n-b)}{a} \tag{1}$$

The wavelet transform function constructed in this paper is a multilevel two-dimensional discrete wavelet transform that captures detailed features such as edges and textures of an image. By multilevel decomposition of the input image, the wavelet transform can separate the different frequency components of the image and better describe the details and structure of the image. Its good scalability enables it to handle image inputs of arbitrary size without the need for a fixed input size.

Firstly, the wavelet transform is performed on the image in the vertical (Y-axis) and horizontal (X-axis) directions to obtain the low-frequency components (L, LL) and high-frequency components (H, LH) of the image in the vertical and horizontal directions, respectively. Then multilevel wavelet decomposition is performed based on the RGB channels of the image respectively. After one level of wavelet decomposition for each channel, more levels of wavelet decomposition are performed on the obtained low-frequency components. Subsequently, all the low-frequency and high-frequency components obtained from the decomposition are combined into a tensor as the output. Finally, the output shape of the wavelet transform is defined, containing the output shape at each level for the application of convolutional neural networks.

Furthermore, to better demonstrate the mathematical principles of the wavelet transform, the wavelet transform function proposed in this paper will be derived in the following through detailed mathematical formulas:

One-dimensional wavelet transform. In one-dimensional wavelet transform, it is assumed that there exists a signal $s(t)$, which is processed by a low-pass filter $g[n]$ and a high-pass filter $h[n]$. After the filtering operation, the result is usually down-sampled to obtain the low and high-frequency parts separately. The formulas are as follows:

$$y_{low}[k] = \sum_n s[2k-n] \cdot g[n] \tag{2}$$

$$y_{high}[k] = \sum_n s[2k-n] \cdot h[n] \tag{3}$$

Two-dimensional wavelet transform. For two-dimensional signals (e.g., images), the wavelet transform is performed first in the vertical direction and then in the horizontal direction. Consider the image $I(x,y)$: First, a one-dimensional discrete wavelet transform (1D-DWT) is applied to each column, producing a low-frequency component (L) and a high-frequency component (H) in the vertical direction. Then, 1D-DWT is applied to each row of the resulting data. This process yields approximate bands of low frequencies in both directions (LL), and three detail components: horizontal high-frequency (LH), vertical high-frequency (HL), and high frequencies in both directions (HH). These sub-bands capture different orientations and scales of the original image, useful for multi-resolution analysis.

Fig 2 below explains the decomposition levels of a two-dimensional discrete wavelet transform (DWT), where "a" (approximation) represents (LL), "v" (vertical detail) represents (LH), "h" (horizontal detail) represents (HL), and "d" (diagonal detail) represents (HH).

a) **Vertical** (column processing): Apply a one-dimensional wavelet transform to each column.

Column approximation (L) and column detail (H):

$$L(x,y)[k] = \sum_n I(x, 2k-n)[y] \cdot g[n] \tag{4}$$

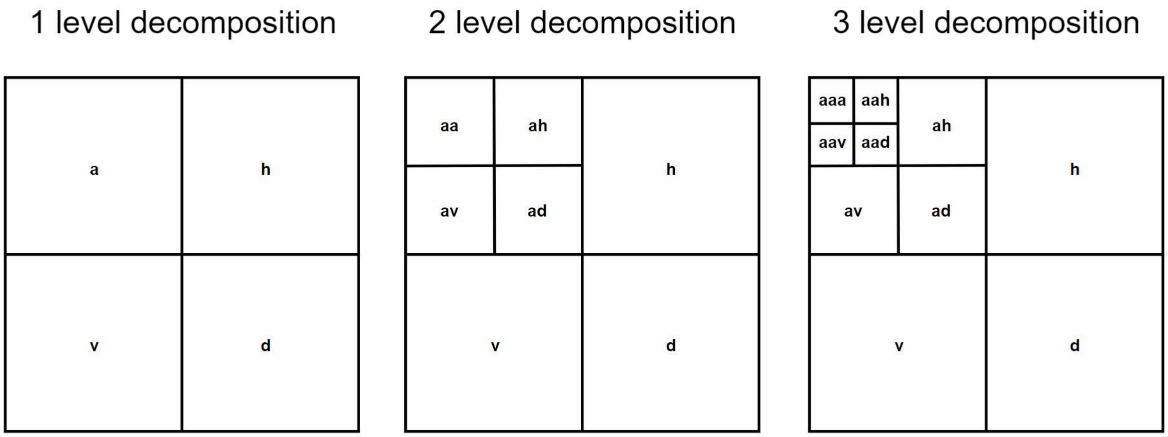

Fig 2. Decomposition process of two-dimensional wavelet transform.

https://doi.org/10.1371/journal.pone.0318530.g002

$$H(x,y)[k]=\sum_{n}I(x,2k-n)[y]\cdot h[n] \tag{5}$$

b) **Horizontal** (row processing): Apply 1D wavelet transform to the results of column processing L and H.

Row approximation and row detail:

$$LL(x,y)[k]=\sum_{m}L(2k-m,y)[x]\cdot g[m] \tag{6}$$

$$LH(x,y)[k]=\sum_{m}L(2k-m,y)[x]\cdot h[m] \tag{7}$$

$$HL(x,y)[k]=\sum_{m}H(2k-m,y)[x]\cdot g[m] \tag{8}$$

$$HH(x,y)[k]=\sum_{m}H(2k-m,y)[x]\cdot h[m] \tag{9}$$

c) **Recursive decomposition**:

In wavelet decomposition, an image is decomposed into sub-bands of different frequency ranges through repeated applications of low-pass and high-pass filtering, which represent the various frequency components of the original image. After obtaining the four sub-bands: (LL, LH, HL and HH), further recursive decomposition of the LL sub-band is performed to capture finer features in the image. This process decomposes the image into progressively smaller sub-bands, each representing increasingly detailed frequency information.

Taking the n layer wavelet transforms as an example, the recursive decomposition of the four sub-bands can be expressed respectively as:

$$LL_n(x,y)=\sum_{m_1,m_2}LL_{n-1}(2x-m_1,2y-m_2)[x]\cdot g[m_1]\cdot g[m_2] \tag{10}$$

$$LH_n(x,y)=\sum_{m_1,m_2}LL_{n-1}(2x-m_1,2y-m_2)[x]\cdot g[m_1]\cdot h[m_2] \tag{11}$$

$$HL_n(x,y)=\sum_{m_1,m_2}LL_{n-1}(2x-m_1,2y-m_2)[x]\cdot h[m_1]\cdot g[m_2] \tag{12}$$

$$HH_n(x,y)=\sum_{m_1,m_2}LL_{n-1}(2x-m_1,2y-m_2)[x]\cdot h[m_1]\cdot h[m_2] \tag{13}$$

where $g[m]$ are the coefficients of the low-pass filter, $h[m]$ are the coefficients of the high-pass filter, and $n-1$ denotes the LL component of the previous layer.

In practical image processing, only the case of continued decomposition from the LL component is considered usually, as it contains most of the image information. The higher frequency information contained in the rest components will lose important information upon further decomposition.

Representation

The purpose of this module is to extract features from the preprocessed images and obtain the feature map through the convolution and Max-pooling layers.

Classification

This module aims to represent the main features of vehicles in network images and transfer them to target surveillance images, which consists of two classifiers, including a domain classifier and a vehicle type classifier. The domain classifier is used to recognize the data source (web or surveillance), whereas the vehicle type classifier is used to classify the vehicle types in the surveillance images to solve the domain adaption problem [23]. The whole architecture will implement the transfer learning task from the source domain (web) to the target domain (surveillance). Moreover, the attention layer will be used for the vehicle type classification, calculating the weights of all features in the data and extracting the main features from the source data.

In the field of computer vision, three types of attention domains are currently popular, including spatial domain [24], channel domain [25], and mixed domain [26]. Among them, the typical representatives of the mixed domain attention mechanism are the DANet proposed by Fu et al. [26] and the CBAM attention module proposed by Woo et al. [27]. DANet adds two types of attention modules on top of the traditional extended fully connected layer. The location focus module selectively aggregates features at each location by weighting features of all locations, regardless of distance, where similar features are related to each other. The channel attention module selectively emphasizes interdependent channel maps by integrating relevant features from all channel maps, which ultimately further improves the feature representations of the outputs of the two attention modules and contributes to more accurate results.

CBAM combines traditional spatial and channel attention mechanisms. After obtaining the output results through the convolutional layer, the weighted results are first generated by the channel attention module, and then the results are weighted by the spatial attention module. In the above process, CBAM achieves adaptive feature optimization by multiplying the attention map with the input feature map in two independent dimensions (channel and space). In this paper, the mixed domain attention module will be applied in the vehicle type classifier to add weights to the feature map for each channel and evaluate each channel to get the score. The changed scores are then multiplied by the weights of the spatial module to enhance the important features and weaken the trivial features. Fig 3 depicts the structure of CBAM, which is calculated as follows:

$$M_c(F) = \sigma\Big(MLP\big(AvgPool(F)\big) + MLP\big(MaxPool(F)\big)\Big) = \sigma\Big(W_1\big(W_0\big(F^c_{avg}\big)\big) + W_1\big(W_0\big(F^c_{max}\big)\big)\Big) \tag{14}$$

where σ denotes the sigmoid function, $W_0 \in R^{C/r*C}$, and $W_1 \in R^{C*C/r}$. The inputs share the MLP weights: W_0 and W_1, and the ReLU activation function follows W_0.

$$M_s(F) = \sigma\Big(f^{7*7}\big(\big[AvgPool(F); MaxPool(F)\big]\big)\Big) = \sigma\Big(f^{7*7}\big(\big[F^s_{avg}; F^s_{max}\big]\big)\Big) \tag{15}$$

where σ denotes the sigmoid function, and f^{7*7} represents a convolution operation with a filter size of 7*7.

As we can see in Table 2. Structurally [28], SE Net focuses on channel attention by compressing the information globally through the “Squeeze” phase, and then the “Excitation” phase generates the weights of each channel through the fully connected layer, which is used to realign the feature map. However, the SE module only focuses on the channel dimension, ignoring information in the spatial dimension, and the global average set in its squeeze module is too simple to capture complex global information.

RA Net, on the other hand, is a cascaded network of attention modules combining channel and spatial attention, each module consisting of an attention part and a residual part. The

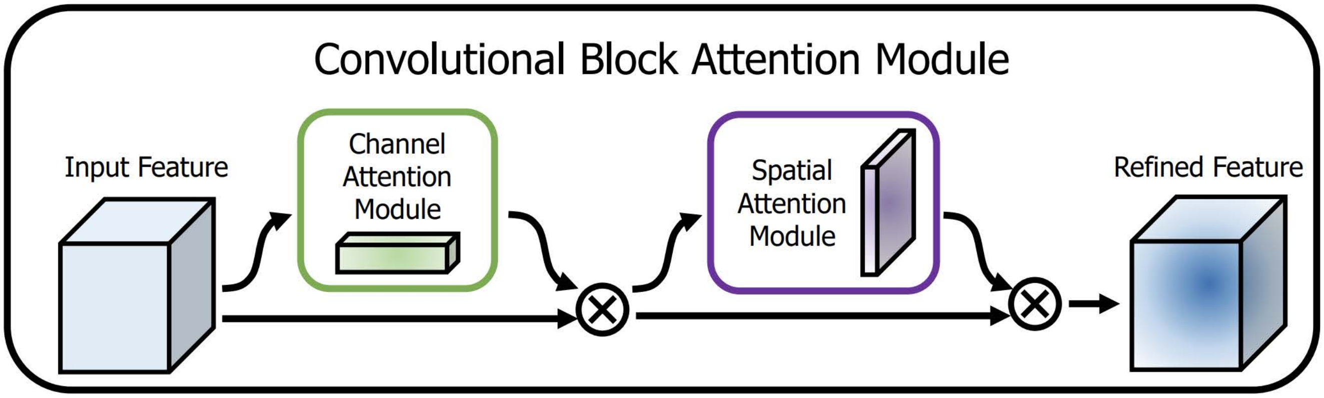

Fig 3. CBAM mechanism [27].

https://doi.org/10.1371/journal.pone.0318530.g003

Table 2. Comparison of different attention mechanisms.

Attention mechanism	Structure	Range
SE Net	Global average pooling -> MLP -> Sigmoid	(0, 1)
RA Net	Top-down network -> Bottom-down network -> 1*1Convs -> Sigmoid	(0, 1)
DA Net	a) Spatial: self-attention in spatial dimension<br>b) Channel: self-attention in channel dimension<br>c) Fuse two branches	(0, 1)
**CBAM**	a) Channel: global pooling in channel dimension -> Conv -> Sigmoid<br>b) Spatial: global pooling in spatial dimension -> MLP -> Sigmoid	(0, 1)

https://doi.org/10.1371/journal.pone.0318530.t002

attention part is adaptively weighted to the input through the mask generation process, and the residual part preserves the original features to ensure gradient flow. However, the proposed bottom-up, top-down structure fails to utilize global spatial information and the introduction of multiple attention modules increases the computational overhead and training complexity significantly.

The DA Net and CBAM modules have a similar structure, with spatial attention and channel attention encapsulated into two separate modules in both networks. The difference is that CBAM generates channel attention through global average pooling and global maximum pooling, and then generates spatial attention through convolutional operations, whereas DA Net employs a self-attention mechanism to generate channel attention and spatial attention, and uses both in parallel. DA Net captures long-range dependencies through non-local operations, which is lacking in CBAM. However, compared with CBAM, DA Net consumes too many resources and has high computational complexity when processing high-resolution images. Therefore, in this paper, a lightweight CBAM module is chosen to minimize computational and resource overheads while maintaining performance.

Table 3 lists the technology used in this paper.

Design and implementation of the DWAN model

This paper is to implement multilevel Discrete Wavelet Transform (DWT) to decompose the input image. Therefore, in the experiment, different functions are defined to divide the input images into odd and even columns, and then the mean (L) and absolute difference (H) are calculated to achieve the wavelet transform in the vertical direction, followed by transposing the input

Table 3. Technology.

**Software**	Framework	PyTorch
	Language	Python
	Version management plan	Git repository
**Hardware**	CPU	Intel(R) Core (TM) i5-10210U CPU @ 1.60GHz 2.11 GHz
	GPU	NVIDIA GeForce GTX MX250

https://doi.org/10.1371/journal.pone.0318530.t003

image and flipping it left and right to realize the wavelet transform in the horizontal direction. After that, three channels (RGB) of the input image are extracted and the first level wavelet transform is performed for each channel. Then the second, third, and fourth-level wavelet transforms are performed for low-dimensional features, and the transform results are stored separately. Finally, the output shape of each wavelet decomposition level is defined to ensure that the wavelet decomposition results of the model at different levels are correctly passed.

Within the model, customized wavelet transform functions are embedded into the input module of the model and the wavelet transformed input features are passed to the various branches of the model. Each branch module contains a convolutional layer with a kernel size of 3x3 and uses a ReLU activation function to introduce nonlinearities so that the model learns the complex nonlinear relationships in the data. A batch normalization layer, which normalizes the activations of the previous layer, is added after each convolutional layer to improve the stability of subsequent training and to speed up the training process. The final added max-pooling layer reduces the spatial dimensionality of the input by down-sampling the maximum value of each 2 x 2 region of the input. Also, a He-normal initializer is used in the convolutional layer to initialize the weights. With a branching module of the form described above, the model can learn hierarchical features and move from simple features in earlier layers to more complex and abstract features in deeper layers.

After the input features have passed through each branching module, the feature mappings obtained from each branching module will be merged through the merging layer. CBAM will process each branch as well as the merged features. In each branch module, the channel attention module (CAM) is applied after the max-pooling layer, while the Spatial Attention Module (SAM) will be used to retain key information in the space at different levels when the branch modules representing different levels of wavelet transform are merged sequentially. The combination of the two forms the CBAM that maximizes the extraction of the key features of the target object.

To explain the intrinsic mechanism of the model in more depth, this study will describe the key layers in the model at the mathematical level:

- **Convolutional layer:**

$$y_{i,j}^{(l)} = \sigma\left(\sum_{m,n} W_{m,n}^{(l)} \cdot x_{i+m,j+n}^{(l-1)} + b^{(l)}\right) \tag{16}$$

where $x^{(l-1)}$ is the input to layer $l-1$, $W^{(l)}$ is the convolution kernel, $b^{(l)}$ is the bias term, σ is the activation function (ReLU), and $y^{(l)}$ is the output feature map.

- **Batch normalization:**

$$\check{x}^{(k)} = \frac{x^{(k)} - \mu B}{\sqrt{\sigma_B^2 + \epsilon}} \tag{17}$$

$$y^{(k)} = \gamma^{(k)} \check{x}^{(k)} + \beta^{(k)} \tag{18}$$

where $x^{(k)}$ is the input, μB and σ_B^2 are the mean and variance of the batch, respectively. $\in$ is a small number that can be divided by zero, γ and β are the learning parameters, and $y^{(k)}$ is the output.

- **Channel attention mechanism:**

The channel attention can express the initial attention weights through global average pooling:

$$CA = \sigma\left(Dense\left(ReLU\left(Dense\left(\frac{1}{H \times M}\sum_{i,j} x_{i,j}\right)\right)\right)\right) \tag{19}$$

Two layers of fully connected networks (Dense) are used here to learn the importance of each channel from the results of global average pooling, and σ is a Sigmoid function to ensure that the outputs are between 0 and 1.

- **Spatial attention mechanism:**

The spatial attention is processed by summarizing the input feature map and then using a small convolution kernel:

$$SA = \sigma\left(Conv\left(Concat\left[AvgPool(x), MaxPool(x)\right], k\right)\right) \tag{20}$$

where $Concat$ is the merge along the channel direction and k is the convolution kernel size.

- **Global average pooling:**

$$y_c = \frac{1}{H \times M}\sum_{i=1}^{H}\sum_{j=1}^{W} x_{i,j,c} \tag{21}$$

where $x_{i,j,c}$ is the value of the feature mapping channel c at position (i, j), and y_c is the global average for channel c.

Based on the formulas for the key layers, the processing of the individual sub-bands in the wavelet transform model can then be derived. For each sub-band I_{Ln}, it is first processed through a convolutional layer:

$$C_{Ln} = \sigma\left(W \cdot I_{Ln} + b\right) \tag{22}$$

Then batch normalization is performed:

$$BN_{Ln} = \gamma\left(\frac{C_{Ln} - \mu}{\sqrt{\sigma^2 + \in}}\right) + \beta \tag{23}$$

Next, after merging the sub-bands, M_{1234} will be obtained, and the merged M_{1234} after additional convolution and batch normalization, will be feature weighted by the spatial attention mechanism:

$$A = \sigma\left(W_A \cdot M_{1234} + b_A\right) \tag{24}$$

$$S = \sigma\left(W_S \cdot A + b_S\right) \tag{25}$$

After spatial attention weighting, the output will be reduced in dimension by global average pooling and connected to the fully connected layer for classification:

$$y_c = \frac{1}{H \times M} \sum_{i=1}^{H} \sum_{j=1}^{W} S_{i,j,c} \tag{26}$$

$$p_k = \frac{e^{y_{ck}}}{\sum_{i=1}^{K} e^{y_{ci}}} \tag{27}$$

where z_k is the k th element of the vector z, p_k is the corresponding classification probability, and K is the total number of categories. SoftMax ensures that all p_k sum to 1 and that each p_k has a value between 0 and 1.

Fig 4 presents the architecture of the constructed model network.

Implementation and results

Testing and evaluation plan

The following metrics will be used to assess the performance of the model. Each metric is expressed mathematically below:

$$Accuracy = \frac{TN + TP}{TN + TP + FN + FP} \tag{28}$$

$$Loss = -\sum_{i=0}^{C-1} y_i \log(p_i) \tag{29}$$

$$Specificity = \frac{TN}{TN + FP} \tag{30}$$

$$Precision = \frac{TP}{TP + FP} \tag{31}$$

$$Sensitivity / TPR / Recall = \frac{TP}{TP + FN} \tag{32}$$

$$F_1 = \frac{2TP}{2TP + FP + FN} = \frac{2(Precision * Recall)}{Precision + Recall} \tag{33}$$

$$FPR = \frac{FP}{FP + TN} \tag{34}$$

A comparative experiment will be conducted based on these metrics to analyze the advantages and disadvantages of the proposed model with other existing models. To evaluate the proposed model, it is first necessary to train the model and record the correctness and loss values of training and validation sets during the model training process as a way to determine if there are any problems with the model structure and parameters. Besides, the model needs to be evaluated multidimensionally and fine-tuned using various types of

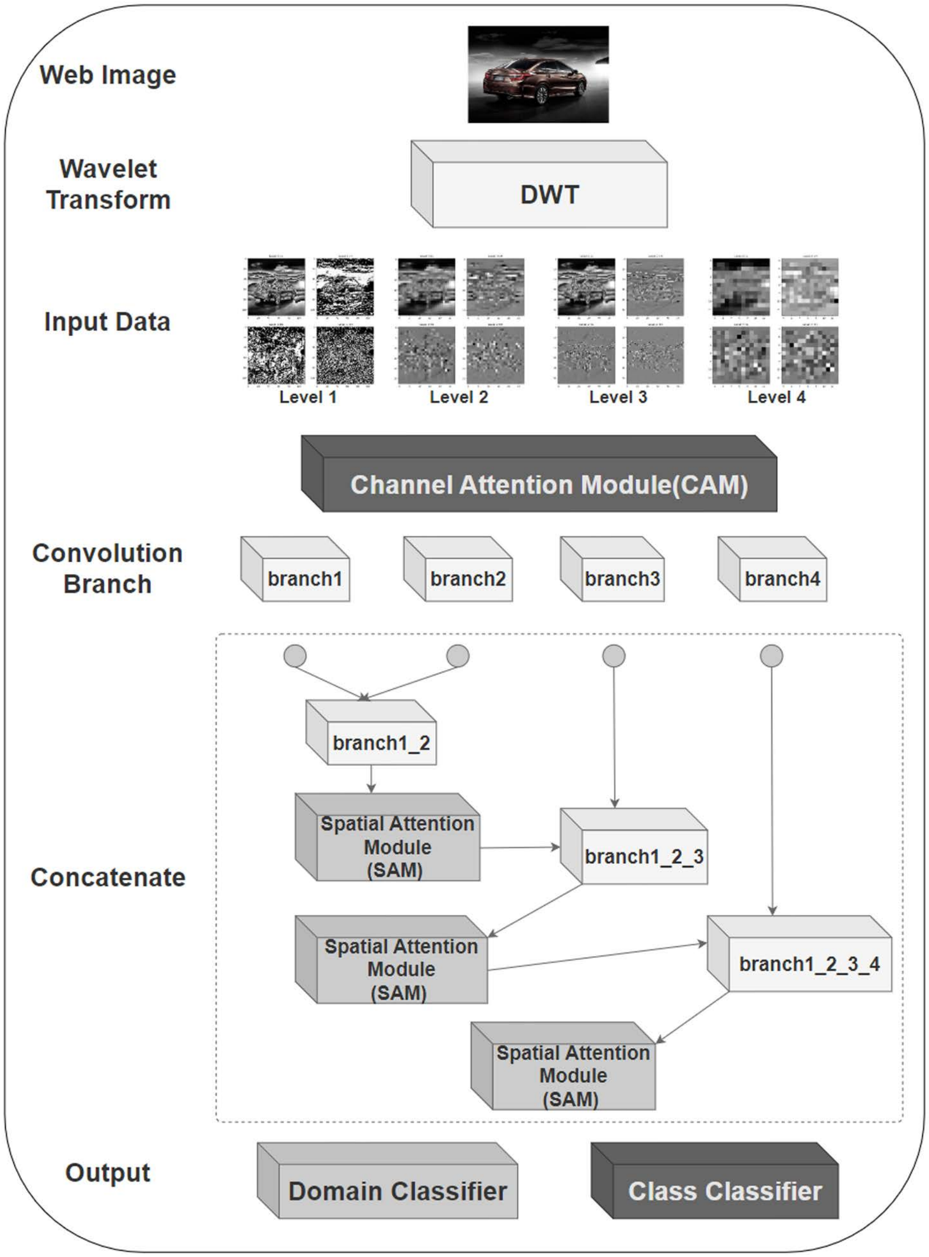

Fig 4. DWAN architecture.

https://doi.org/10.1371/journal.pone.0318530.g004

graphs such as ROC-AUC curves, Precision-Recall curves, and confusion matrices. In this case, the precision rate is the proportion of all samples predicted by the model to be positive examples that are actually positive examples, reflecting the credibility of the model's results in predicting positive examples. Recall, also known as sensitivity or true instance rate, is the proportion of samples that are correctly predicted to be positive by the model out of all samples that are actually in the positive category, reflecting the model's ability to capture samples in the positive category. The F1 score, on the other hand, is a reconciled average of precision and recall, and is intended to synthesize the performance of the two and provide a balanced metric.

Additionally, non-parametric tests will be used to assess the performance of vehicle classification models, which can more accurately and reliably determine differences in model performance across groups or a single group under different occasions, particularly where data do not satisfy specific distributional assumptions and outliers are present, which can contribute to a more holistic understanding and improvement of model performance. The Kruskal-Wallis H test is suitable for comparing median differences between multiple independent groups. In contrast, the Friedman test compares groups with dependent measurements, such as multiple measurements of a signal group or subject under different conditions.

Kruskal-Wallis test:

$$H' = \frac{H}{1 - \frac{\sum_{j=1}^{G}\left(t_j^3 - t_j\right)}{N^3 - N}} \tag{35}$$

where H' is the test statistic in the presence of repeated ordinal times, G is the number of groupings of different tied ranks, and t_j is the number of tied values within group i that are tied at a particular value.

Friedman test:

$$Q = \frac{12}{nk(k+1)}\sum_{j=1}^{k} R_j^2 - 3n(k+1) \tag{36}$$

where Q is the test statistic, n and k are the number of subjects and treatment conditions, respectively. And R_j is the rank sum of the jth treatment condition.

Experiments

In this section, the data preprocessing and DWAN model optimization steps are presented, as well as the model classification results for the source domain (web data) and the target domain (surveillance data) separately. In addition, the proposed DWAN model is directly compared with other neural network architectures (e.g., ResNet, MobileNet, etc.) under the same training setup, environment, and dataset.

Experimental design. Experimental with the DWAN model will start by preprocessing the CompCars dataset, observing the performance of the model once it has used the data, and determining the selection of data in the dataset accordingly. In the next step, the residual mixed domain attention mechanism module and the GoogleNet-based Inception module will be combined with the original DWAN model, and these two DWAN models will be critically compared with the original DWAN to evaluate their final performance. Finally, the LIME algorithm will be used to visually interpret the DWAN model, and the results will be analyzed to adjust the specific training methodology to obtain the best results.

Dataset. This paper will utilize the dataset from the article named "A Large-Scale Car Dataset for Fine-Grained Categorization and Verification" on CVPR 2015. This dataset called Comprehensive Cars (CompCars) includes the images from web and surveillance systems. The web data consists of 1,716 car models, 13786,726 images capturing the entire car, and 27,618 images capturing the car parts while the surveillance data contains 50,000 images captured in the front view. Each car model is labeled with five attributes, including maximum speed, displacement, number of doors, number of seats, and type of car. The dataset mainly provides usable data for three kinds of computer vision tasks: vehicle type detection, fine-grained detection, and attribute prediction.

However, the dataset contains too much data volume and categories of vehicle images from web and surveillance sources for the equipment and cloud servers used in this work to carry such a large volume of data. Therefore, 21,540 and 3,798 images were selected from each of the web and surveillance data and divided into five categories. These include MPV, sedan, hatchback, pickup, and sports. In the experiment, I will divide the web images into training (85%) and validation (15%) sets to test the performance of the model on the source domain (web) data. Then the source domain data will be used as the training set and the target domain (surveillance) data will be used as the test set to show the model's ability to solve the domain adaptation problem and the transferability effect.

In addition, to further validate the generalizability and robustness of the model, the proposed model in this paper experimented on the vehicle image datasets containing multiple types of vehicles from Kaggle's datasets named "Stanford Car Body Type Data" and "Types of Car Image Dataset", which contains 10 categories with 8144 images and 6 categories with 16502 images respectively.

Performance results using the web dataset. To better accomplish the vehicle type classification task, the vehicles were first classified into 12 categories from the CompCars dataset with a total of 90,539 images (Fig 5). However, as the vehicle samples are too concentrated in hatchbacks, sedans, and SUVs, after a series of filtering and adjusting, some categories with too sparse sample size (convertible, crossover, estate, fastback, hardtop convertible) are selectively and directly deleted, and some samples are randomly removed from the three categories with the largest sample size (hatchback, sedan, and SUV) to make the dataset as balanced as possible (Fig 6).

Before performing transfer learning on the model, first of all, the base model containing only labeled classifiers needs to be trained and validated on the source domain data. After organizing the dataset, various preprocessing operations were first performed on the data. Table 4 displays the effect of the model on the web image dataset after adding different preprocessing operations, while Fig 7 presents the difference between the original data and the data after the preprocessing operation.

From the experimental data, it can be seen that when only horizontal flip operation is performed on the dataset, the model's evaluation of the data (accuracy, loss, precision, recall, and F1-score) is lower than the metrics after horizontal rotate, fly rotate, and random pan operations are performed on the dataset. The performance of the model on the dataset is also degraded if too many preprocessing operations are added (e.g., clipping, scaling, and padding).

Unfortunately, it was found during the experiments that the performance of the proposed DWAN model is still insufficient in the seven selected vehicle categories (MPV, SUV, sedan, hatchback, minibus, pickup, and sports), although some of the categories with extremely low sample sizes had been removed in previous work. Thus, to make the dataset more balanced, five categories of vehicle data are finally retained in this paper, including MPV, sedan, hatchback, pickup, and sports. The test results are shown in Table 5, Figs 8 and 9.

From the above test results, it can be seen that the performance of the DWAN model proposed in this study is much higher than that of the seven-classification on the five-classification task, with an accuracy and precision rate of more than 80.00%. Therefore, in this paper, the network architecture of DWAN is redesigned based on the residual module of ResNet and the inception module of GoogleNet, and two variants, Res-DWAN and Inception-DWAN, are obtained respectively.

The advantage of the residual structure is that it prevents the gradient vanishing problem that occurs in deep networks, allowing the network to learn constant mappings that deepen the network and improve performance. After combining the residual structure with the

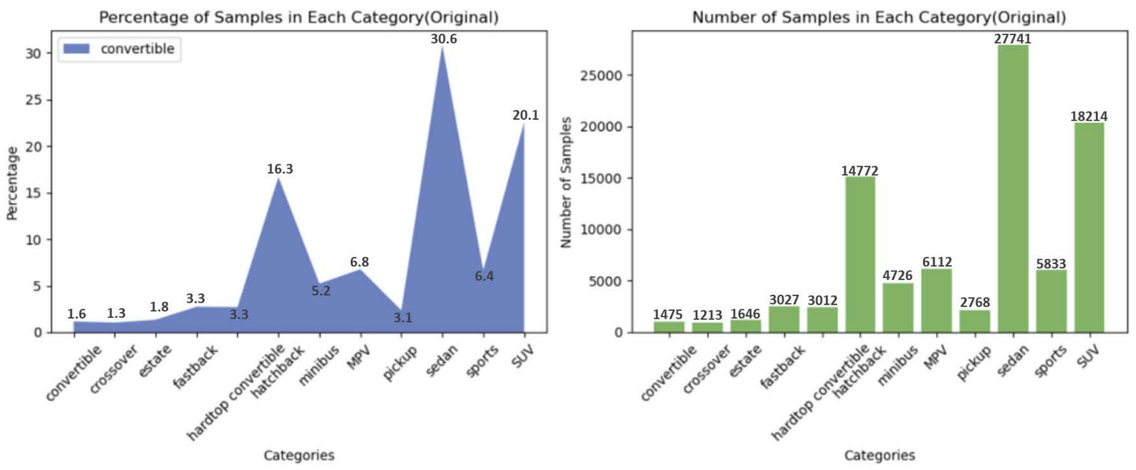

Fig 5. Distribution of original dataset.

https://doi.org/10.1371/journal.pone.0318530.g005

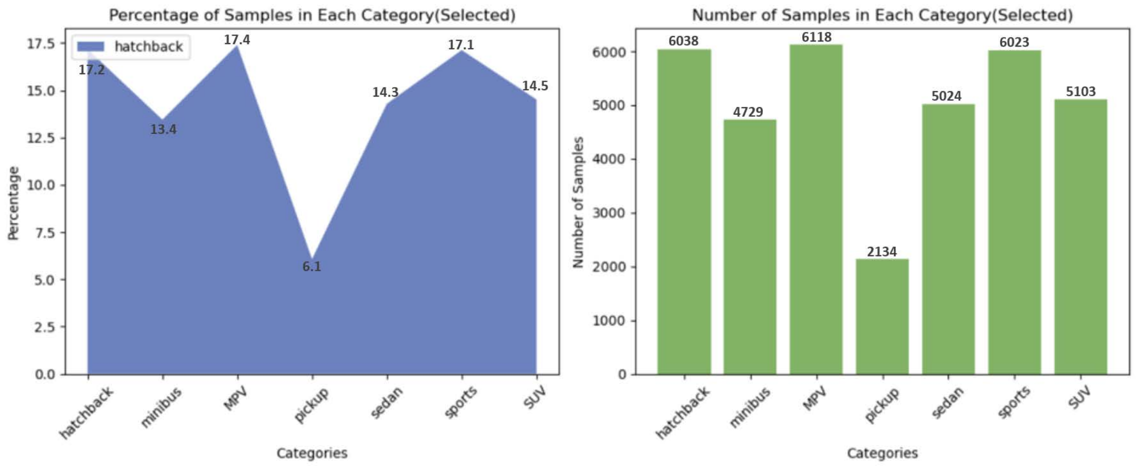

Fig 6. Distribution of selected dataset.

https://doi.org/10.1371/journal.pone.0318530.g006

Table 4. Comparison of different preprocessing operations.

Preprocessing	Accuracy	Loss	Precision	Recall	F1
Only with a horizontal flip	0.63	0.70	0.64	0.63	0.61
Horizontal flip, rotation range, shift range	0.69	0.67	0.67	0.69	0.68

https://doi.org/10.1371/journal.pone.0318530.t004

attention mechanism, this structure not only maintains the advantages of the residual network but also allows the network to make more efficient use of the input features and improves the model's ability to focus on important information.

As can be seen from the above Figs 10 and 11 above, in the designed residual hybrid domain attention mechanism, the CAM module pays attention to the input feature X and connects it to the fully connected layer, obtains two feature vectors, Y1 and Y2, through two

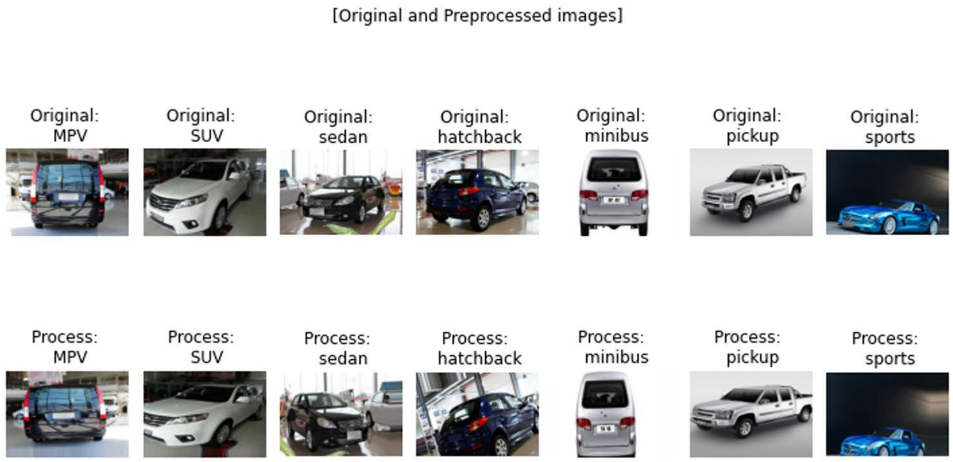

Fig 7. Difference between original and processed data.

https://doi.org/10.1371/journal.pone.0318530.g007

Table 5. Comparison of different categories.

Category	Accuracy	Loss	Precision	Recall	F1
7 classes	0.63	0.70	0.64	0.63	0.61
5 classes	0.74	0.67	0.75	0.72	0.72

https://doi.org/10.1371/journal.pone.0318530.t005

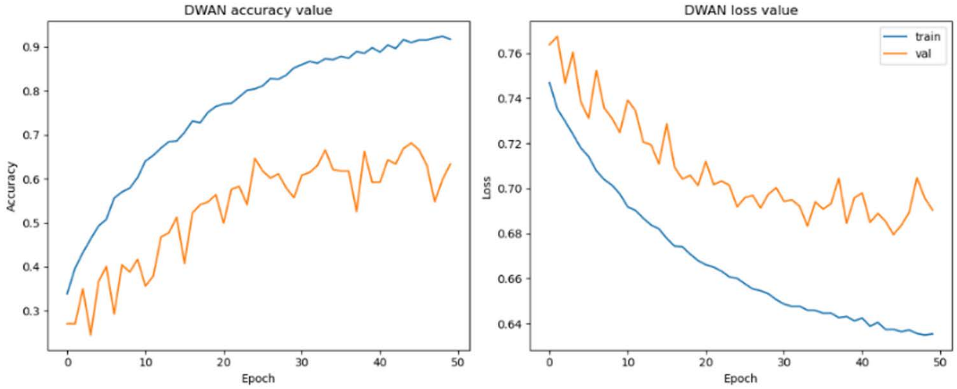

Fig 8. Performance in 7 categories.

https://doi.org/10.1371/journal.pone.0318530.g008

operations, average pooling and max pooling, respectively, and then fuses them into a single feature vector, Y, which represents the importance of each channel, through SoftMax operation. Subsequently, Y is multiplied channel-by-channel with the input features X to strengthen the important feature channels and suppress the unimportant ones. The SAM module, on the other hand, focuses on the importance of spatial location. It receives X as input and generates a spatial attention map Mask, which is multiplied pixel-by-pixel with the input feature X, thereby reinforcing the important spatial locations. Convolutional branches of the residual structure are also added to the newly designed network structure, and the two together form Res-DWAN, a variant based on the original DWAN framework.

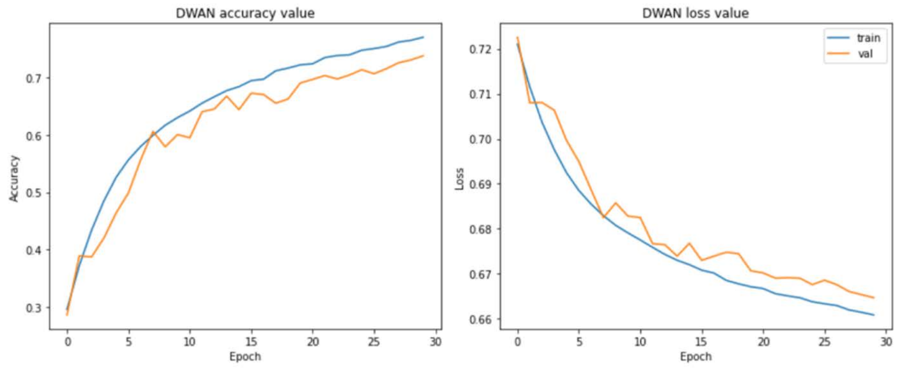

Fig 9. Performance in 5 categories.

https://doi.org/10.1371/journal.pone.0318530.g009

The branch modules of the DWAN model show that the input to each branch module is an image that has been wavelet transformed at four different levels, after which the four modules are spliced sequentially. This network structure is very similar to the Inception module of GoogleNet, so the convolutional branches and subsequent structure of the original DWAN are encapsulated as modules similar to Inception-V1, and the modules are stacked in progressively increasing numbers of channels as the main framework of the Inception-DWAN variant, as depicted in Figs 12 and 13. In addition, the residual hybrid domain attention mechanism mentioned above will also be used in this Inception-DWAN network variant.

Next, the CompCars dataset will be used to determine the optimal hyperparameters for fine-tuning the vehicle classification model. Throughout the experimental phase, systematic adjustments of hyperparameters, including the image size, batch size, learning rate, and dropout rate, are conducted.

The experimentation will employ a univariate approach, where each hyperparameter is modified independently while maintaining the other variables constant, to ascertain its effect on the model's effectiveness, and the updated hyperparameters will be selected for a new round of experiments after each experiment. In this case, the initial setting for the image size is 512, the batch size is 16, the learning rate is 0.0001, and the dropout rate will be 0.5. Through this comprehensive analysis, the most suitable hyperparameters can be identified to optimize the model's vehicle classification performance on the CompCars dataset.

From the experimental results in Table 6 above, it can be seen that increasing the batch size during training leads to a reduction in the variance of the gradient, which directly improves the accuracy of the gradient. However, a smaller batch size will make the gradient change fluctuate more and the network is not easy to converge, while a larger gradient will reduce the number of parameter updates in each round of training, which requires an increase in the number of training rounds and training time. Therefore, as verified by the above experiments, the most appropriate batch size for the model is 16.

The selection of the learning rate has a significant impact on the model as it determines how much (or how fast and how many steps) the model parameters are adjusted at each step of the parameter update. The learning rate also interacts with other aspects of the optimization process, and this effect can be nonlinear. For example, smaller batch sizes are best paired with smaller learning rates, as smaller batch sizes can also be noisy, which requires careful tuning of the parameters, however, a learning rate that is too small can be counterproductive. Therefore, in the experiments, when the batch size is equal to 16, the learning rate of 0.0005 is more suitable, when the model has better loss, accuracy, and callback rate during the training process.

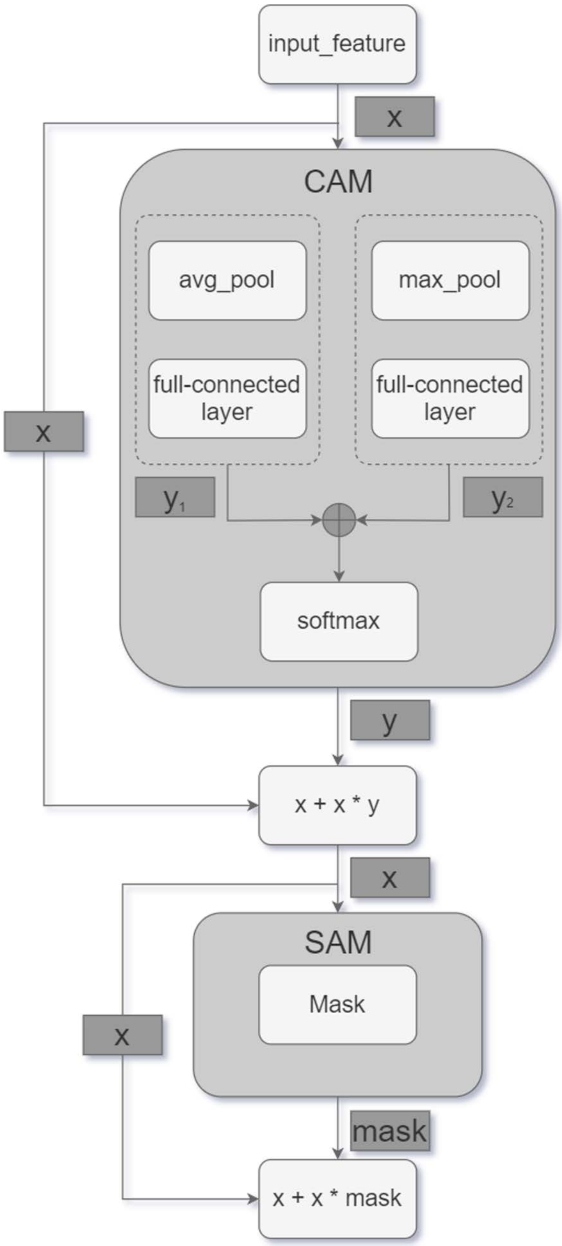

Fig 10. Residual hybrid domain attention mechanism.

https://doi.org/10.1371/journal.pone.0318530.g010

Dropout is a regularization technique employed to prevent the networks from noise and overfitting by randomly dropping units from the neural network during training. If the model is trained on a limited dataset, or if the training data contains lots of noise, then the model might face the problem of overfitting. To combat this, one solution is to reduce the number of hidden units responsible for feature extraction. Dropout essentially deletes or inactivates a portion of the hidden units at each training stage, which helps to prevent the network from becoming too dependent on any single feature. After experiments, the model achieves the optimal accuracy on the CompCars dataset with a dropout of 0.2, suggesting a balance between network complexity and generalization ability.

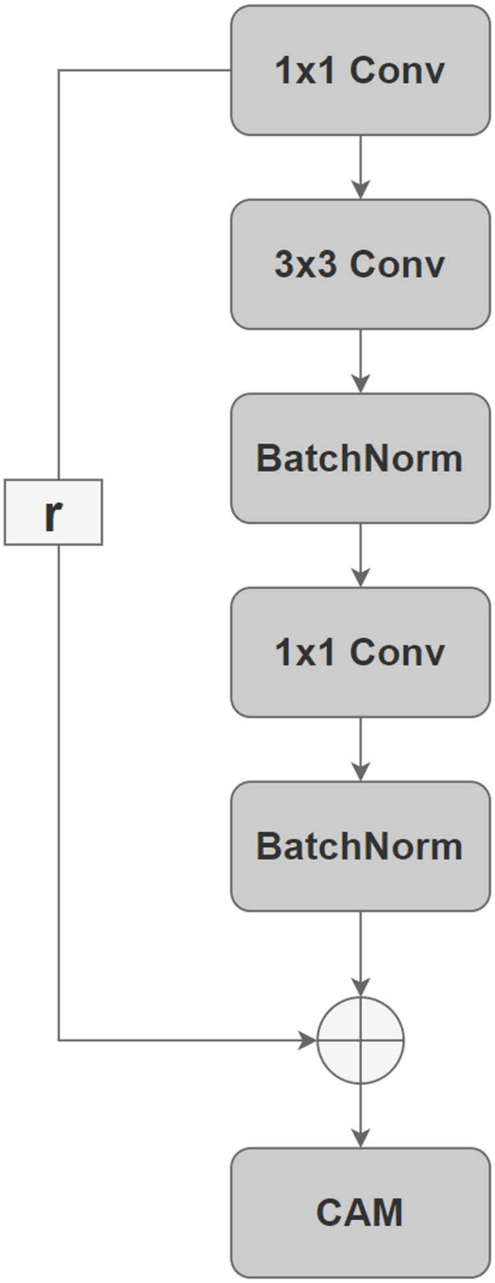

Fig 11. Residual convolutional branch.

https://doi.org/10.1371/journal.pone.0318530.g011

Therefore, the subsequent experiments on the model will be conducted with the same parameters and environment: The figure size is 224, the batch size is 16, the learning rate is 0.0005, the dropout rate is 0.2, and the optimizer is Adam.

As can be seen from the experimental results in the above Figs 14–17 and Tables 7–9, the Res-DWAN structure has significant performance advantages over the original DWAN model. The loss can be narrowed down to a lower value and the accuracy is also substantially improved over the original model. The confusion matrix reveals that the model maintains a low error in all categories after equalizing the samples of the dataset. In addition,

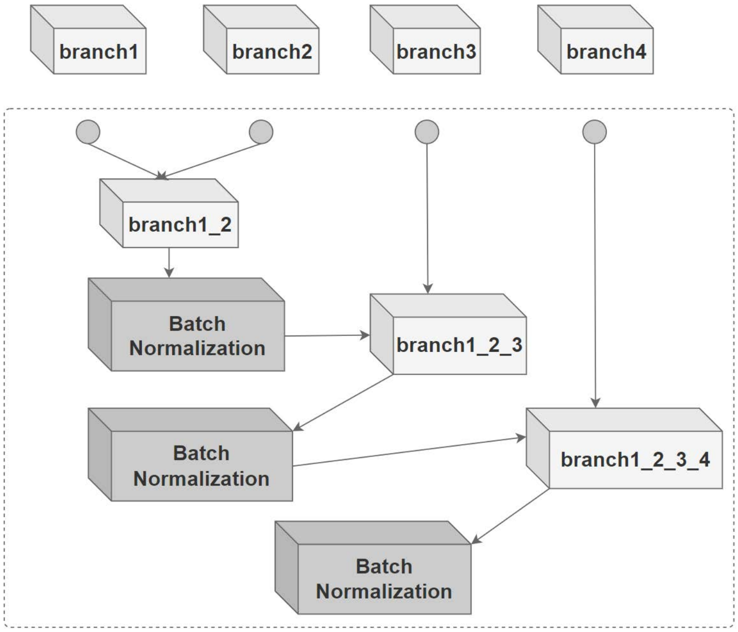

Fig 12. Convolution module.

https://doi.org/10.1371/journal.pone.0318530.g012

the PR and the ROC-AUC curves also show that the model retains high AUC values in all categories.

However, in terms of individual category metrics, the model's performance on individual categories (e.g., hatchbacks) differs markedly from its performance on other categories, with large differences in sensitivity and specificity. This suggests that Res-DWAN is not performing as expected in some specific categories.

When comparing the performance of the three networks, DWAN, Res-DWAN, and Inception-DWAN (Figs 18–21, Tables 10 and 11), Res-DWAN performs the best with an accuracy of 0.82, a loss rate of 0.53, as well as precision, recall and F1-score of 0.82, demonstrating superior balanced performance and strong generalization. In contrast, DWAN has lower performance metrics, while Inception-DWAN has a slight advantage in recall (0.77), suggesting that it may be more effective in recognizing positive classes under specific conditions.

Additionally, the accuracy and loss plots of Res-DWAN clearly show a stable improvement in performance and a reduction in loss during training, indicating better learning efficiency and stability of the model. Its confusion matrix reveals high-precision classification decisions, especially excellent recognition ability of certain categories, while the precision-recall and ROC-AUC curves further confirm its excellent classification ability and good true-positive rate. On the other hand, the confusion matrix and classification reports of Inception-DWAN indicate that although the model performs well in the recognition of specific categories, it still suffers from misclassification in certain categories, which is also reflected in the precision-recall curve, showing the challenge of balancing recall and precision. However, it can also be noticed from the above figures that the Inception-DWAN structure has not yet reached its optimal performance during training, and the subsequent addition of more training rounds along with tuning of the hyperparameters will make it a more promising model to classify specific types of vehicles.

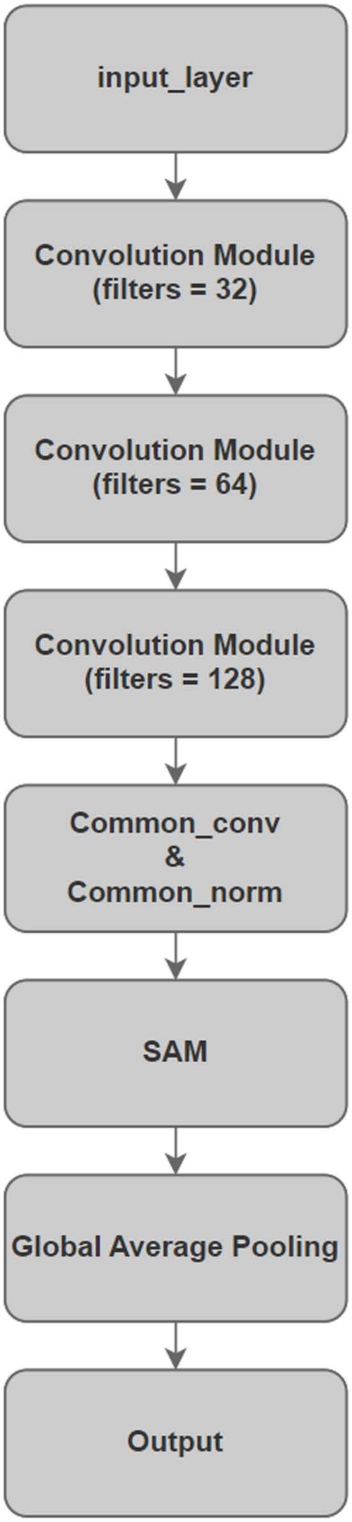

Fig 13. Inception-DWAN architecture.

https://doi.org/10.1371/journal.pone.0318530.g013

Table 6. Evaluation results of Res-DWAN model with different parameters.

Parameters				Accuracy	Loss	Precision	Recall	F1
Figure size	Batch size	Learning rate	Dropout					
512	16	0.0001	0.5	0.81	0.55	0.79	0.80	0.80
224	16	0.0001	0.5	0.81	0.53	0.83	0.82	0.82
224	16	0.0005	0.5	0.82	0.55	0.80	0.81	0.81
224	16	0.0005	0.2	0.83	0.50	0.85	0.83	0.83
224	32	0.0005	0.2	0.82	0.66	0.82	0.83	0.82

https://doi.org/10.1371/journal.pone.0318530.t006

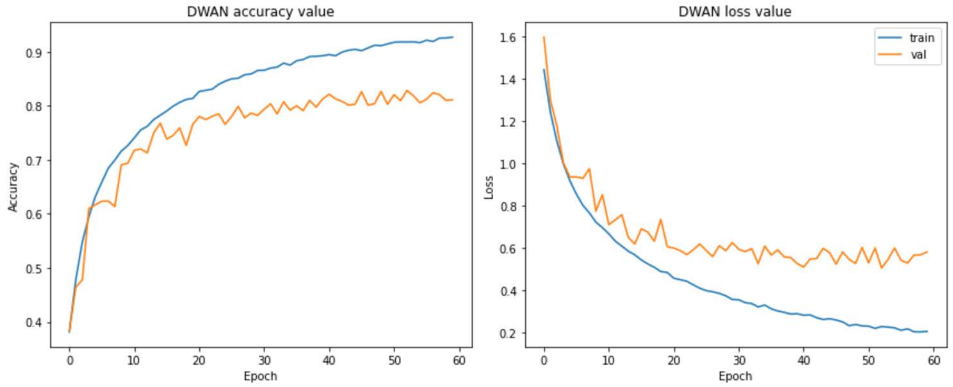

Fig 14. Accuracy and loss of Res-DWAN.

https://doi.org/10.1371/journal.pone.0318530.g014

The above Fig 22 demonstrates a comparison of the accuracy of three different model variants in the vehicle classification task, with the significance of the differences assessed using the Kruskal-Wallis H-test. The H-statistic of 12.54 corresponds to a p-value of 0.0019, which indicates that the difference in accuracy between the different model variants is statistically significant. The results show that the Res-DWAN model significantly improves classification performance.

Lime (Local Interpretable Model-Agnostic Explanations) is an algorithm that uses trained local agent models to interpret individual samples. It is assumed that for a black-box model that needs to be interpreted, the instances of interest are first sampled, new sampling points are generated in their vicinity by perturbation and the predictions of the black-box model are obtained, and then an interpretable model (e.g., neural network) is trained using the new dataset to obtain a good local approximation of the black-box model. Fig 23 shows the results of feature visualization using the Lime method for different categories of sample instances, from which it can be seen that the proposed model can effectively capture the heterogeneous features of different types of vehicles. For example, in the hatchback image of Category 2 and the pickup truck image of Category 3, the main feature captured by the model is the main outline of the vehicle.

However, it is obvious that the background of the images can be greatly influenced by the image features extracted by the model. For example, in the MPV image of category 0 and the sedan image of category 1, the surrounding scene of the vehicle is focused on by the model, which affects the quality of the extracted features. Whereas in the sports car image of category 4, it is more evident that the model focuses on parts of the background that are similar in color to the vehicle, which affects the performance of the model on the dataset.

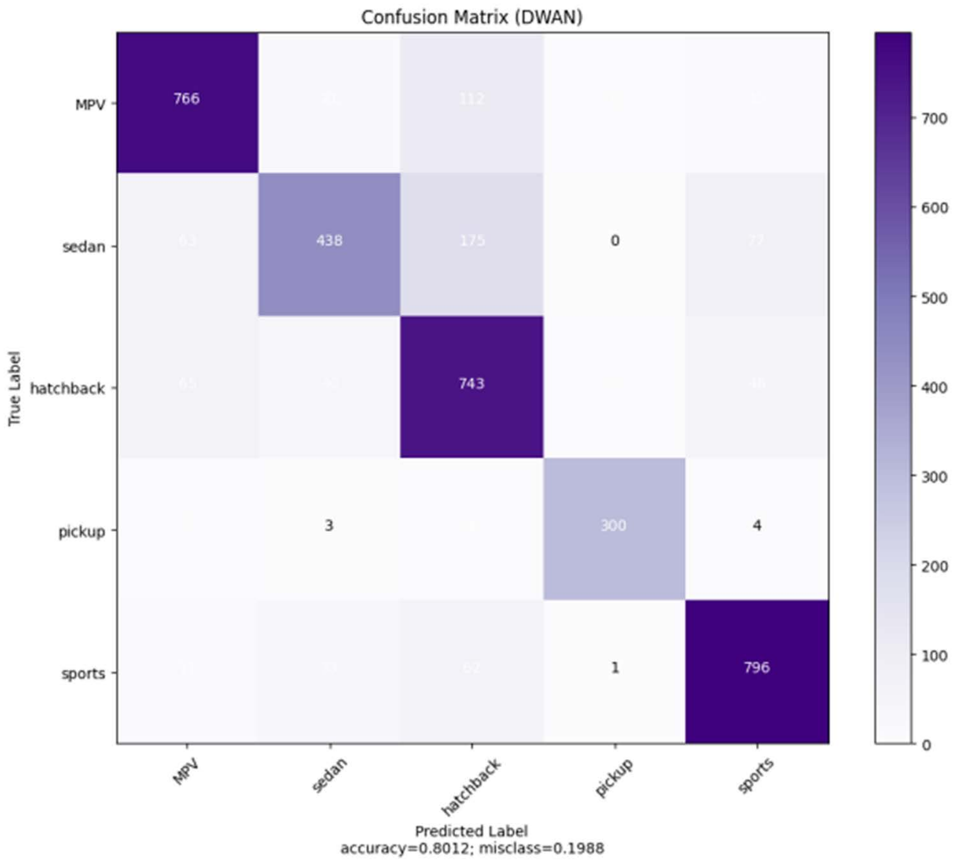

Fig 15. Confusion matrix of Res-DWAN.

https://doi.org/10.1371/journal.pone.0318530.g015

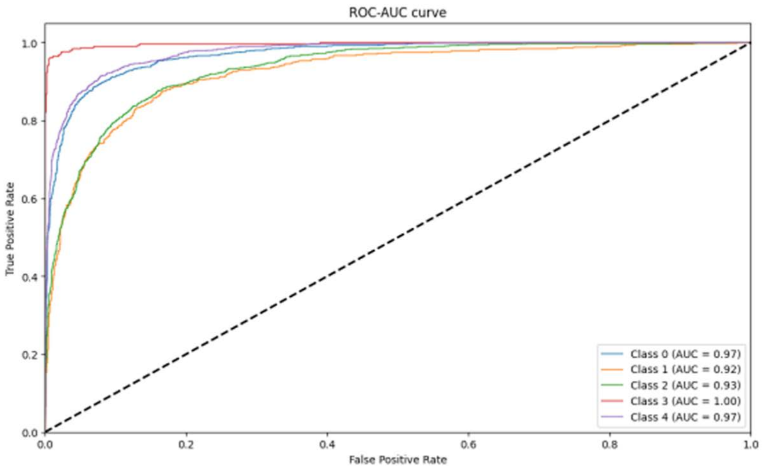

Fig 16. ROC-AUC curve of Res-DWAN.

https://doi.org/10.1371/journal.pone.0318530.g016

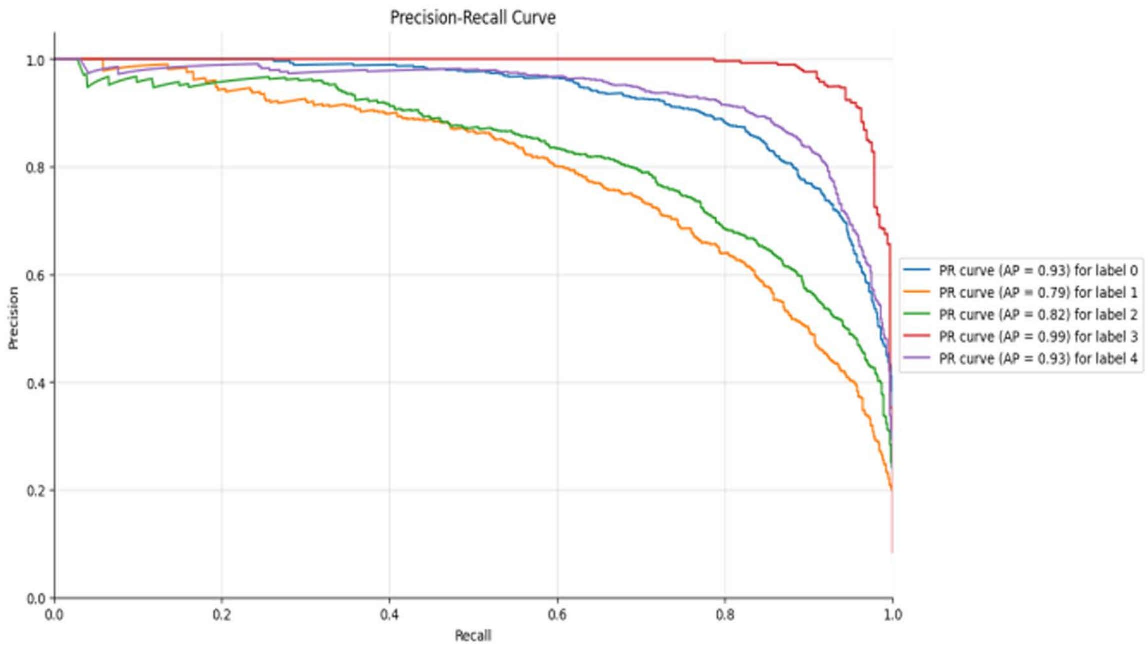

Fig 17. Precision-Recall curve of Res-DWAN.

https://doi.org/10.1371/journal.pone.0318530.g017

Table 7. Comparison of different network variants.

Model	Accuracy	Loss	Precision	Recall	F1
DWAN	0.74	0.67	0.75	0.72	0.72
Res-DWAN	0.82	0.53	0.82	0.80	0.81
Inception-DWAN	0.75	0.65	0.76	0.77	0.76

https://doi.org/10.1371/journal.pone.0318530.t007

Table 8. Classification report of Res-DWAN.

	Precision	Recall	F1	Sample size
MPV (0)	0.84	0.83	0.84	917
sedan (1)	0.81	0.57	0.67	753
hatchback (2)	0.67	0.82	0.74	905
pickup (3)	0.94	0.93	0.94	320
sports (4)	0.84	0.87	0.85	930
accuracy			0.79	398
macro avg	0.82	0.80	0.81	3798
weighted avg	0.80	0.79	0.79	3798

https://doi.org/10.1371/journal.pone.0318530.t008

To demonstrate the effect of the attention mechanism on the model's ability to extract image features, this study employs a class activation map (CAM), which is used to analyze the interpretability of neural networks, to show the classifier's positional weights relative to the data.

As can be seen from the heatmap (Figs 24–26), in the model that does not include any attention mechanism, the model tends to capture global features in the image, resulting in too many dispersed features being captured by the model (in the case of the MPV image). If the

Table 9. Sensitivity specificity of Res-DWAN.

	Metrix	Value
0	Sensitivity	0.887064
1	Specificity	0.972257

https://doi.org/10.1371/journal.pone.0318530.t009

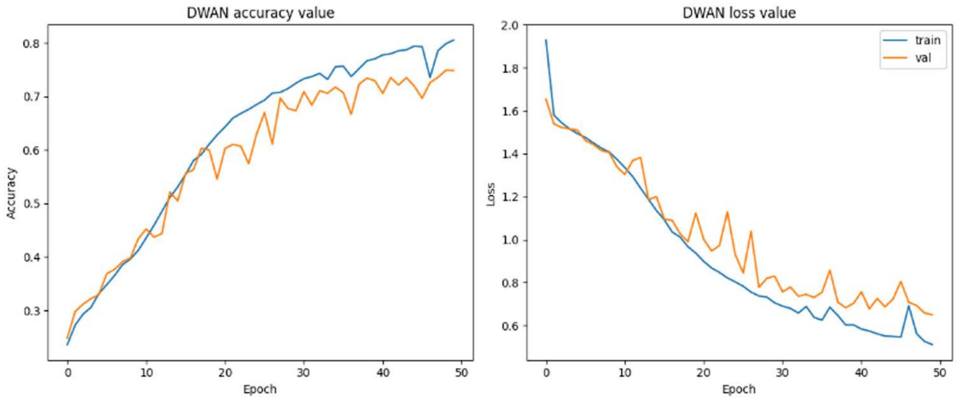

Fig 18. Precision-Recall curve of Inception-DWAN.

https://doi.org/10.1371/journal.pone.0318530.g018

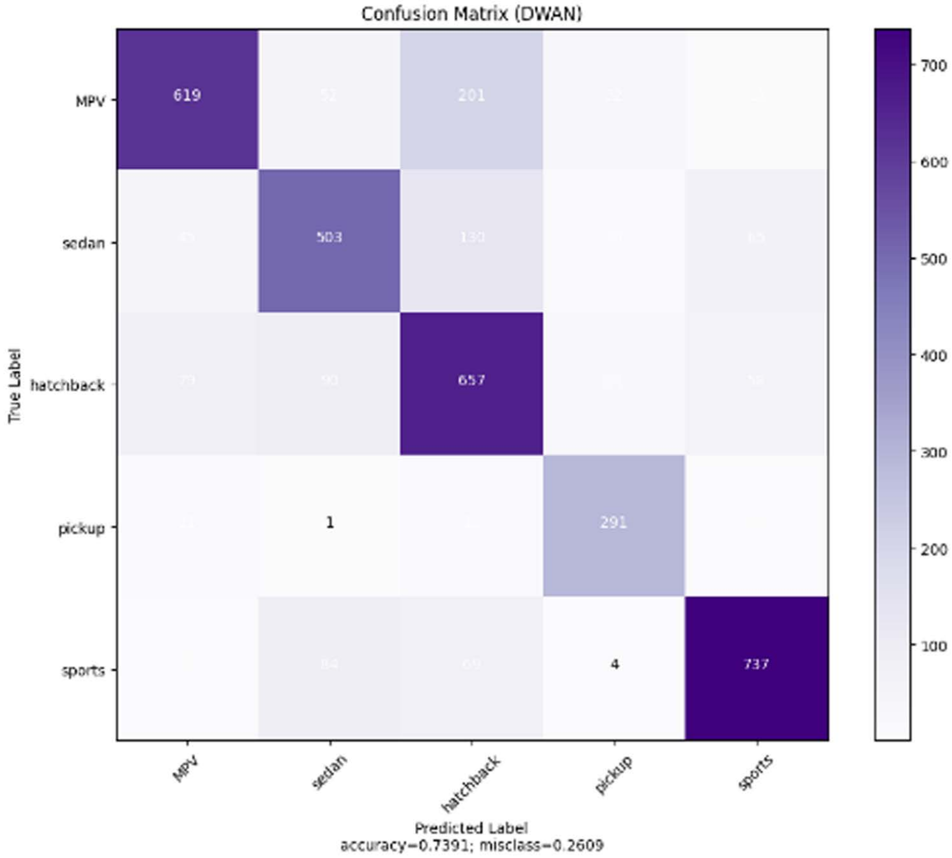

Fig 19. Confusion matrix of Inception-DWAN.

https://doi.org/10.1371/journal.pone.0318530.g019

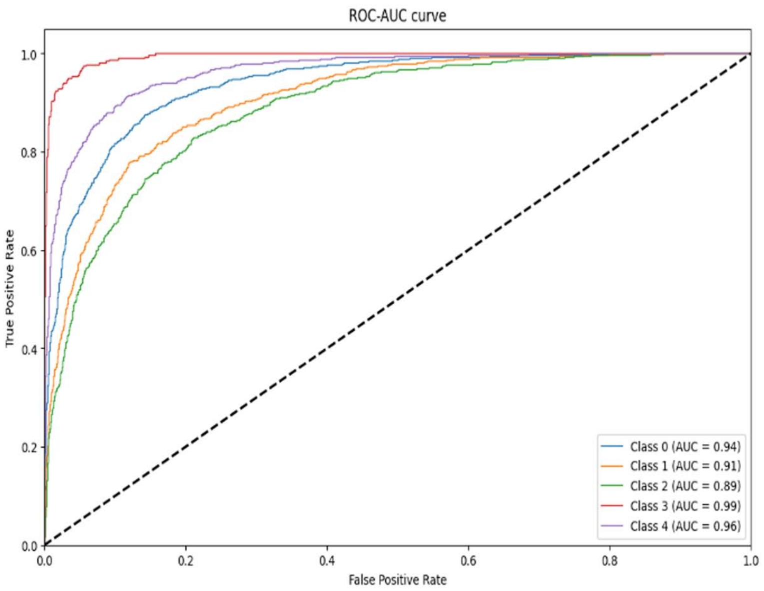

Fig 20. ROC-AUC curve of Inception-DWAN.

https://doi.org/10.1371/journal.pone.0318530.g020

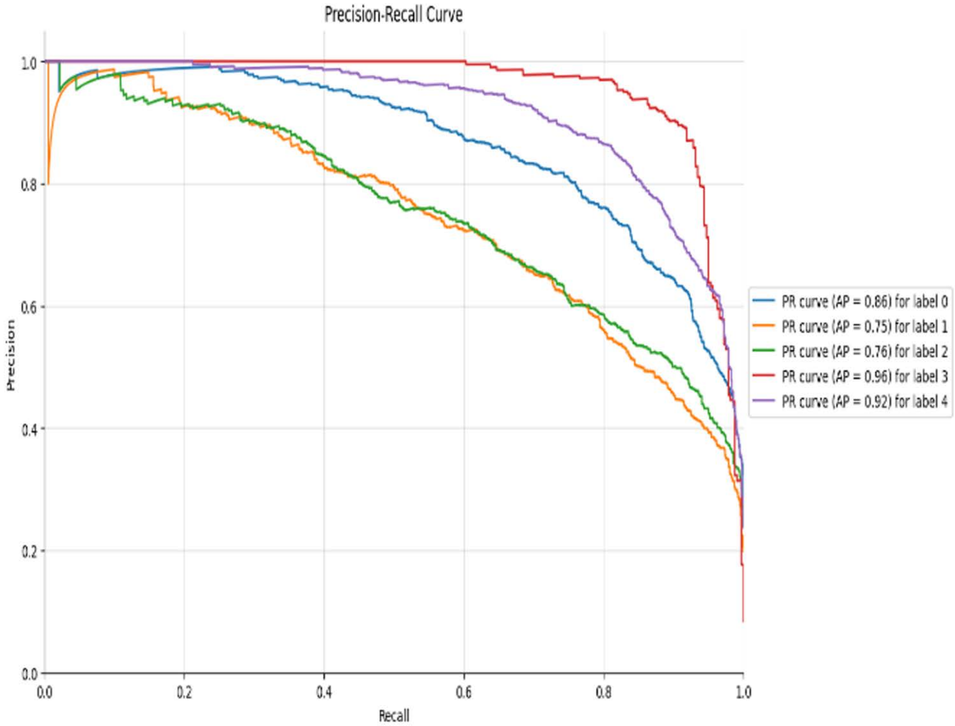

Fig 21. Precision-Recall curve of Inception-DWAN.

https://doi.org/10.1371/journal.pone.0318530.g021

targets in the image are too similar to their background colors, it is difficult for the model to capture the classification targets in the image (in the case of the Sedan image). However, in the model with the addition of the CBAM, it can be observed that the model captures more

Table 10. Classification report of Inception-DWAN.

	Precision	Recall	F1	Sample size
MPV (0)	0.82	0.70	0.76	917
sedan (1)	0.69	0.66	0.67	753
hatchback (2)	0.63	0.73	0.68	905
pickup (3)	0.82	0.93	0.87	320
sports (4)	0.84	0.82	0.83	930
accuracy			0.75	3798
macro avg	0.76	0.77	0.76	3798
weighted avg	0.75	0.75	0.75	3798

https://doi.org/10.1371/journal.pone.0318530.t010

Table 11. Sensitivity specificity of Inception-DWAN.

	Metrix	Value
0	Sensitivity	0.91
1	Specificity	0.91

https://doi.org/10.1371/journal.pone.0318530.t011

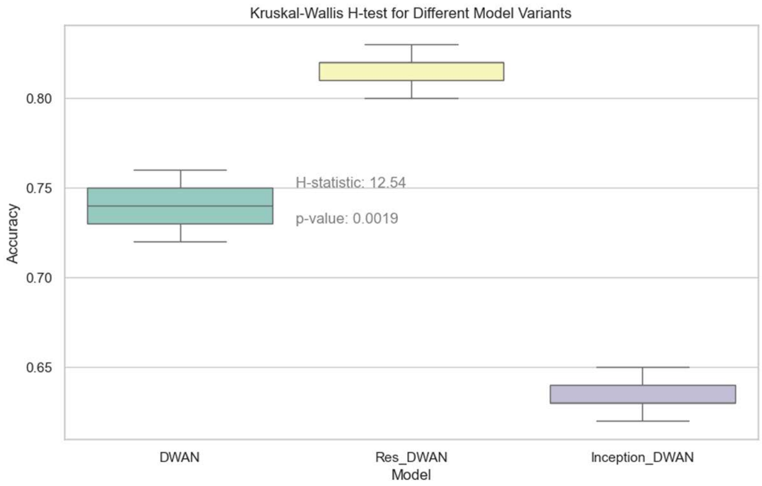

Fig 22. Kruskal-Wallis H-test for different model variants.

https://doi.org/10.1371/journal.pone.0318530.g022

features around the target object in the image. The model will focus on capturing specific parts of the vehicle in the image.

In addition, image size is also a very important factor in this study. When four levels of discrete wavelet transform are applied to an image of size (224, 224), the output feature sizes of different levels are (112, 112, 12), (56, 56, 12), (32, 32, 12) and (16, 16, 12), respectively. The size of the image decreases abruptly after the last two levels of wavelet transform, which can seriously affect the ability of the model to capture important features of the image (Fig 27). The experiment tried to resize the image to (512, 512), but from the experimental results, the indicators of the model did not improve but rather decreased after the image size was adjusted too large. Therefore, subsequent experiments will attempt to reduce the level of wavelet transform as a way to improve the model's ability to capture features.

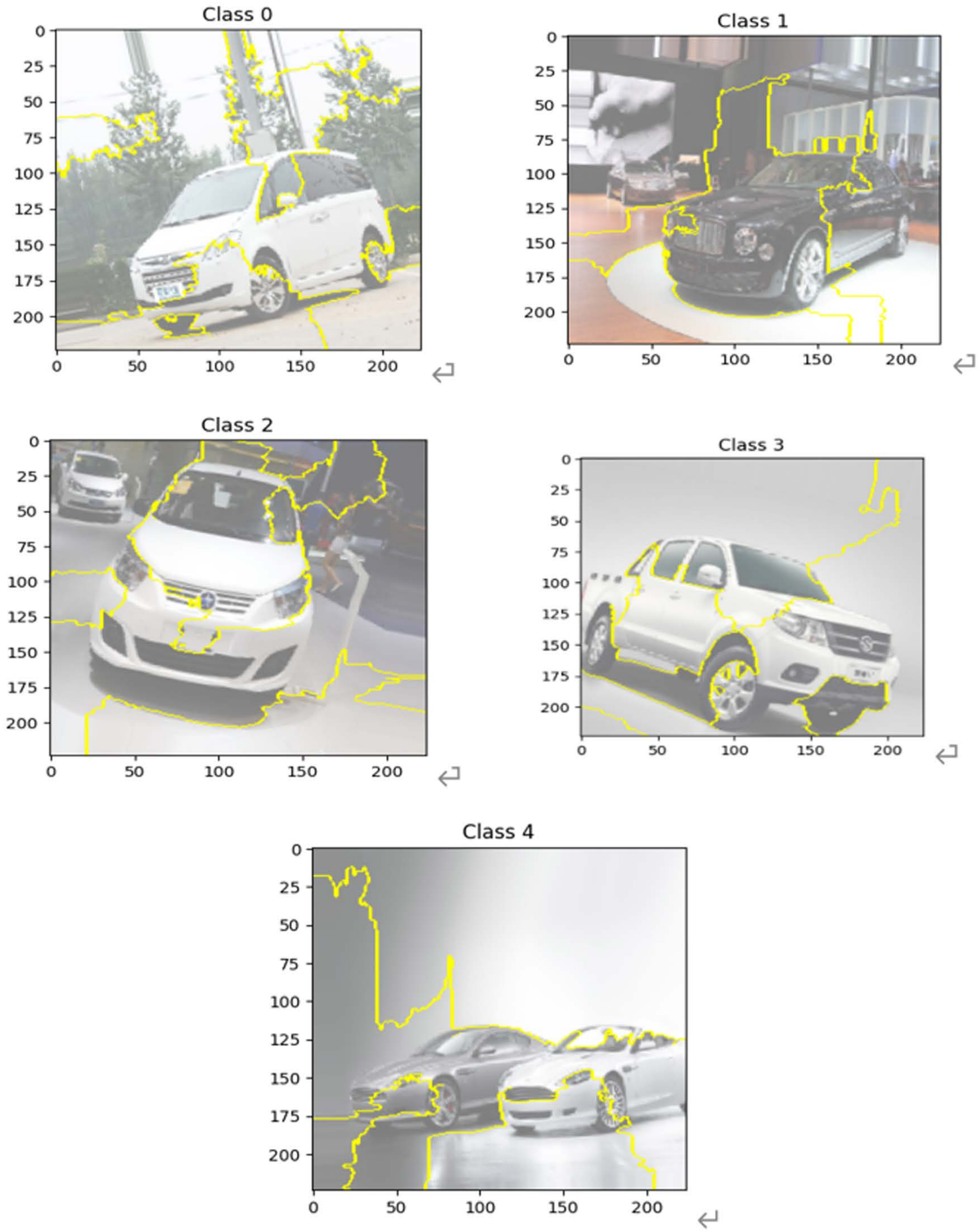

Fig 23. Visualization of model features (Lime).

https://doi.org/10.1371/journal.pone.0318530.g023

As can be seen from Table 12 and Fig 28, the effectiveness of the model on the dataset is significantly improved after reducing the number of levels of the discrete wavelet transform, and there were also significant differences in the distribution of accuracy between levels,

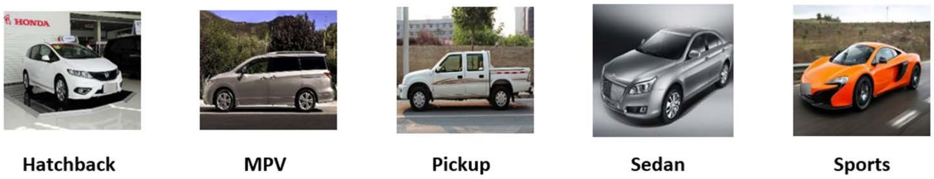

Fig 24. Original images of different vehicles.

https://doi.org/10.1371/journal.pone.0318530.g024

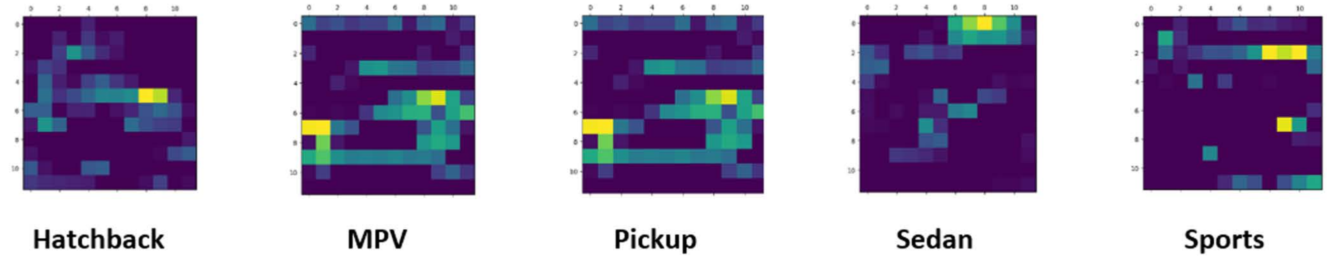

Fig 25. Grad-Cam images of different vehicles.

https://doi.org/10.1371/journal.pone.0318530.g025

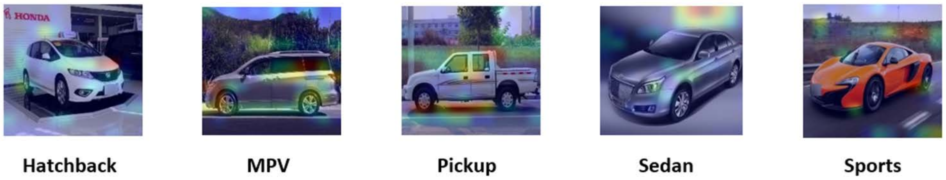

Fig 26. Attentional visualization of different vehicles.

https://doi.org/10.1371/journal.pone.0318530.g026

indicating the level of wavelet transform has a significant effect on the accuracy of the vehicle classification model. The results of Friedman's test show that the difference in accuracy between different wavelet transform levels is statistically significant.

Moreover, it has been experimentally demonstrated that the model's performance is optimized when only two levels of the wavelet transform, (112, 112, 12) and (56, 56, 12), are retained, which no longer capture certain features that are too localized and no longer important. Also, the overhead of the model is significantly reduced after the reduction of excessive wavelet transforms. However, the experimental results also show that as the levels of wavelet transform decrease, the loss of the model during training increases significantly. This proves that the model also omits some important features to some extent when learning features. Therefore, in the subsequent work, when reducing the number of levels of the discrete wavelet transform, the internal structure of the model also needs to be adjusted to reduce the loss of the model during training and validation.

Comparison with using different datasets. As can be seen from Table 13, in all three vehicle datasets containing different categories, the model proposed in this paper shows high performance, and the average accuracy of the proposed model reaches more than 85.00% on all three datasets while keeping the loss low. However, it can also be seen from this experiment

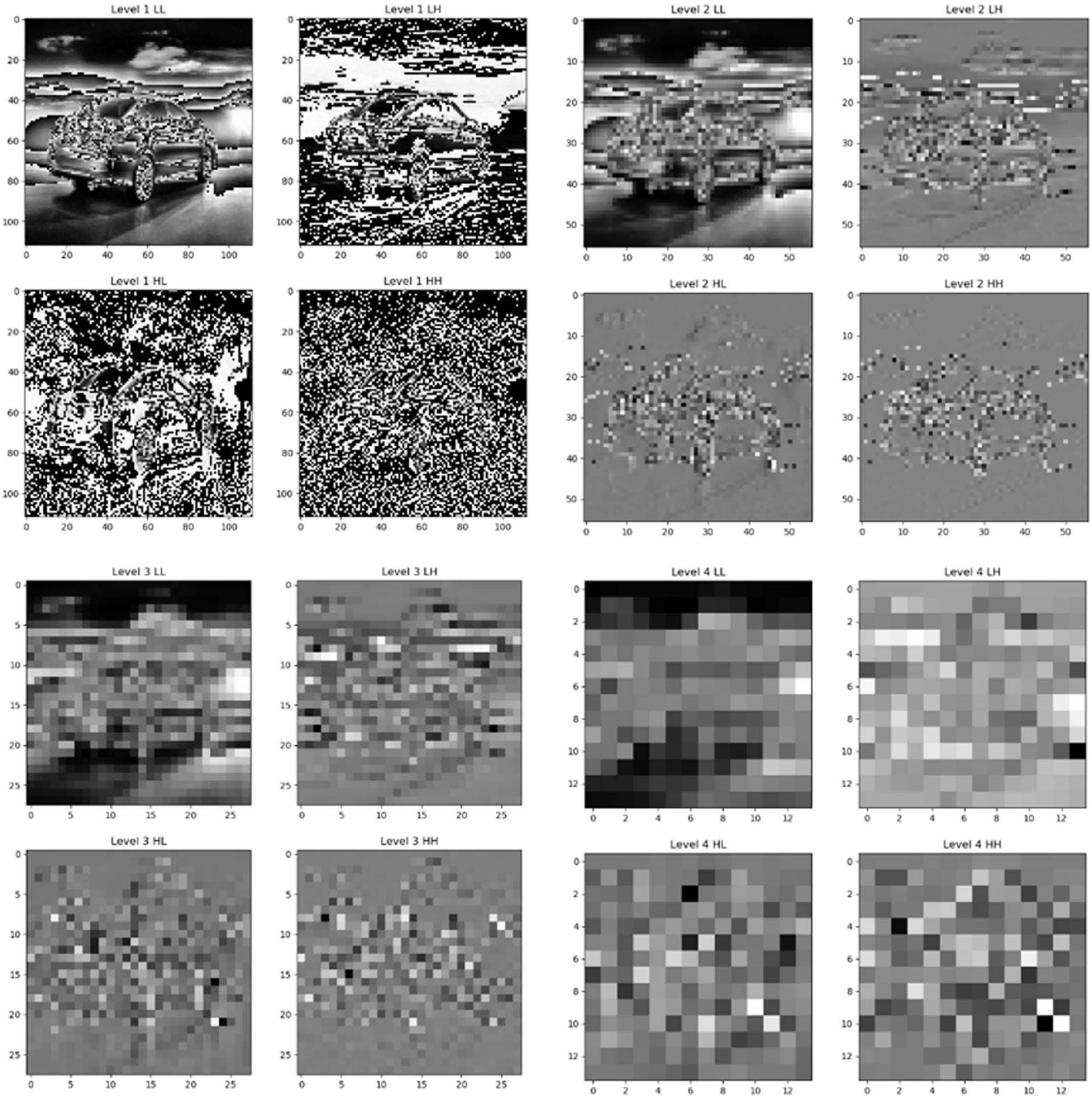

Fig 27. Four levels of discrete wavelet transform.

https://doi.org/10.1371/journal.pone.0318530.g027

that the performance of the proposed model in the dataset with more categories and a small amount of data (Stanford Cars) is slightly inferior to the other two datasets with fewer categories and a large amount of data, especially in terms of accuracy and loss. This is due

Table 12. Evaluation results of DWAN model(base) with different levels of wavelet transform.

Wavelet Transform	Accuracy	Loss	Precision	Recall	F1
2 level	0.88	0.70	0.88	0.87	0.87
3 level	0.85	0.65	0.86	0.86	0.85
4 level	0.83	0.50	0.85	0.83	0.83

https://doi.org/10.1371/journal.pone.0318530.t012

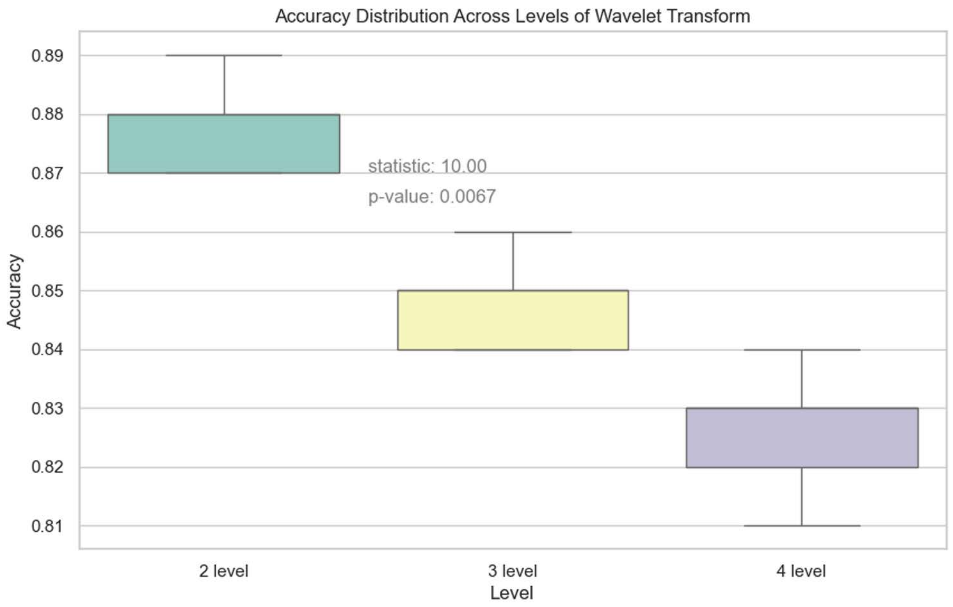

Fig 28. Accuracy distribution across levels of wavelet transform.

https://doi.org/10.1371/journal.pone.0318530.g028

Table 13. Evaluation results of DWAN model (base) with different levels of WT.

Datasets	Vehicle Type	Accuracy	Loss	Precision	Recall	F1
CompCars	Hatchback, Pickup, MPV, Sedan, Sports	0.88	0.70	0.88	0.87	0.87
Stanford Cars	Cab, Convertible, Coupe, Hatchback, Minivan, Other, SUV, Sedan, Van, Wagon	0.83	0.74	0.85	0.84	0.85
Types of Cars Image	Convertible, Coupe, Hatchback, Pickup, SUV, Sedan	0.85	0.43	0.86	0.85	0.85

https://doi.org/10.1371/journal.pone.0318530.t013

to the complex structure of the model itself, which requires more data to be trained for each vehicle category to stimulate the model's performance.

Fair comparison with other deep learning models. In the comparison experiments, five existing pre-trained models, MobileNet, ResNet50, InceptionV3, EfficientNet, and RegNet, are used to compare their performance with the model proposed in this paper under the same conditions: the figure size is 224, the batch size is 16, the learning rate is 0.0005, and the optimizer is Adam. The weights of the layers of the existing pre-trained models are not frozen and the initial weights are set to none to make the initial conditions the same so that the performance of the models can only be compared when using the dataset in this paper.

As can be seen from Table 14, the performance results of the integrated model proposed in this paper are significantly better than the other five existing pre-trained models on all metrics. The next figures will illustrate the effect plots and curves of each model on different metrics.

Table 14. The performance results of comparison with other existing models.

Model	Accuracy	Loss	Precision	Recall	F1
MobileNet	0.55	1.13	0.64	0.60	0.57
ResNet50	0.41	1.48	0.45	0.48	0.40
InceptionV3	0.24	1.56	0.06	0.24	0.09
EfficientNet	0.54	1.23	0.59	0.59	0.51
RegNet	0.40	1.46	0.38	0.40	0.35
**DWAN**	**0.79**	**0.57**	**0.82**	**0.80**	**0.81**

https://doi.org/10.1371/journal.pone.0318530.t014

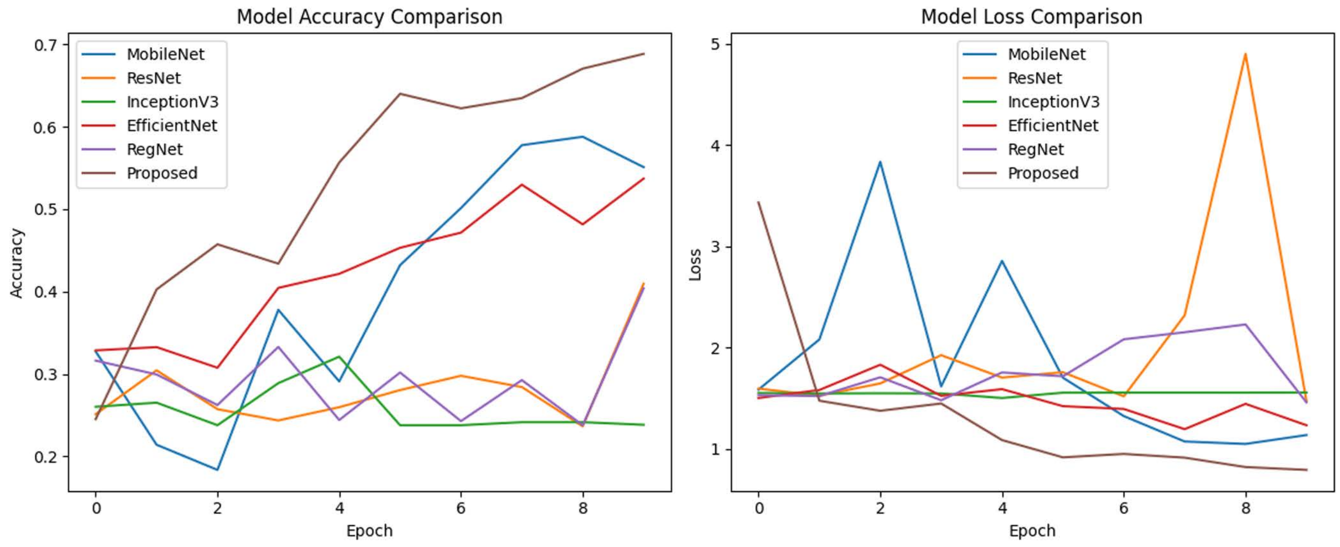

Fig 29. Model accuracy and loss comparison.

https://doi.org/10.1371/journal.pone.0318530.g029

In the performance comparison experiments, it can be seen from the above data (Figs 29–32) on the accuracy, loss, confusion matrix, ROC-AUC curves, and precision-recall curves that the model proposed in this study exhibits superior performance in the testing phase compared to the five widely adopted pre-trained models.

Specifically, in the existing pre-training models, there are significant fluctuations in accuracy and loss during the training process. In contrast, the proposed model shows a trend of rapid convergence of losses during training, while the accuracy increases rapidly and eventually stabilizes at the peak level. Additionally, the metrics of the existing pre-training models are far inferior to the proposed model in all categories of the dataset.

Through the Kruskal-Wallis H test in Fig 33, it is possible to clarify the performance difference between different models in multiple runs. The statistical results show that the proposed model significantly outperforms MobileNet, ResNet50, InceptionV3, EfficientNet, and RegNet in terms of accuracy, which provides strong statistical support for the superior performance of the proposed model.

In order to verify that the proposed model successfully balances receptive field size and computational efficiency, 100 samples from the training set are selected using the same input size (224, 224, 3) and fixed batch size (32), and the time required for each model to process the same sample size is calculated. The results in Table 15 show that the processing speed of the

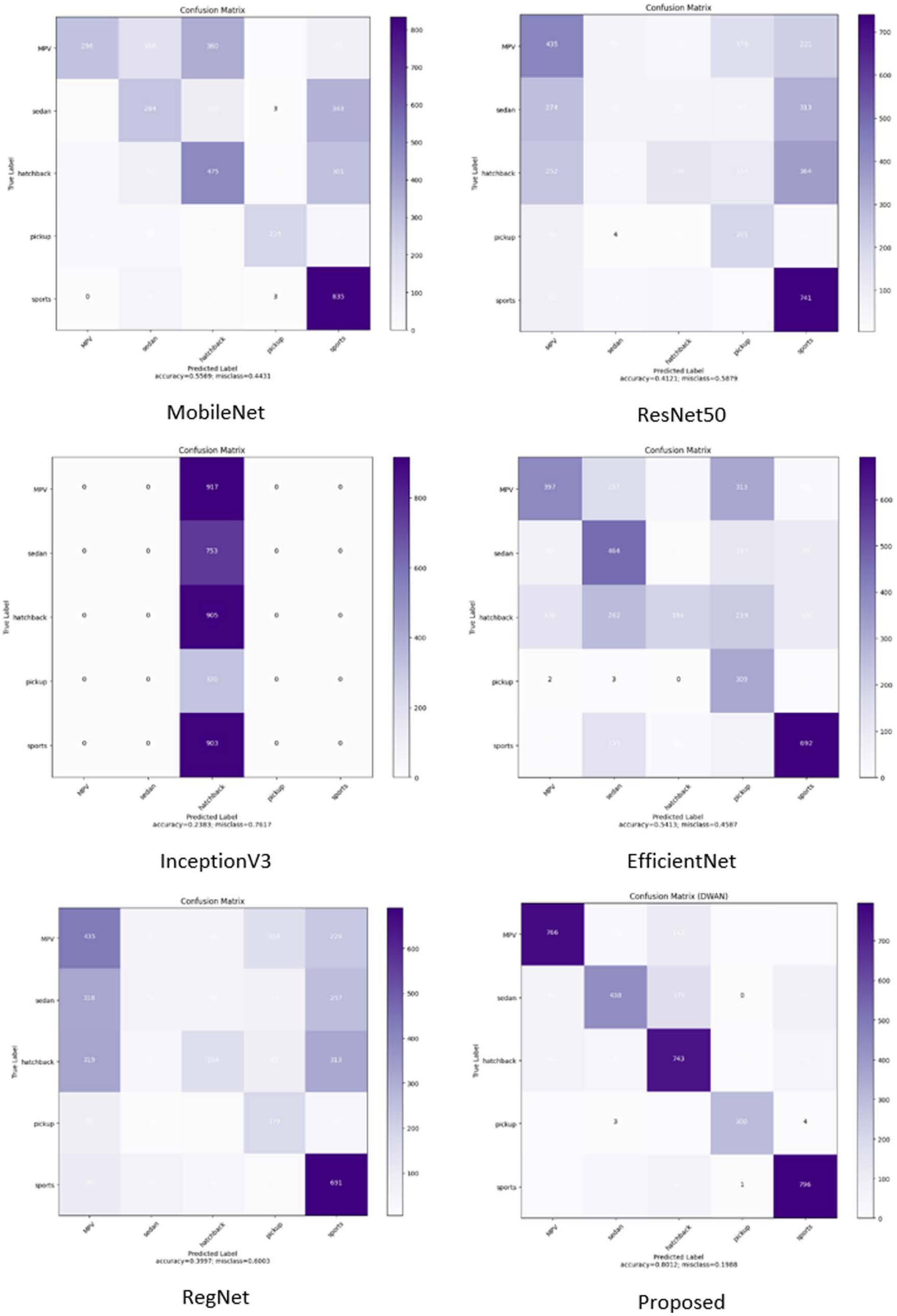

Fig 30. Confusion matrix for comparison.

https://doi.org/10.1371/journal.pone.0318530.g030

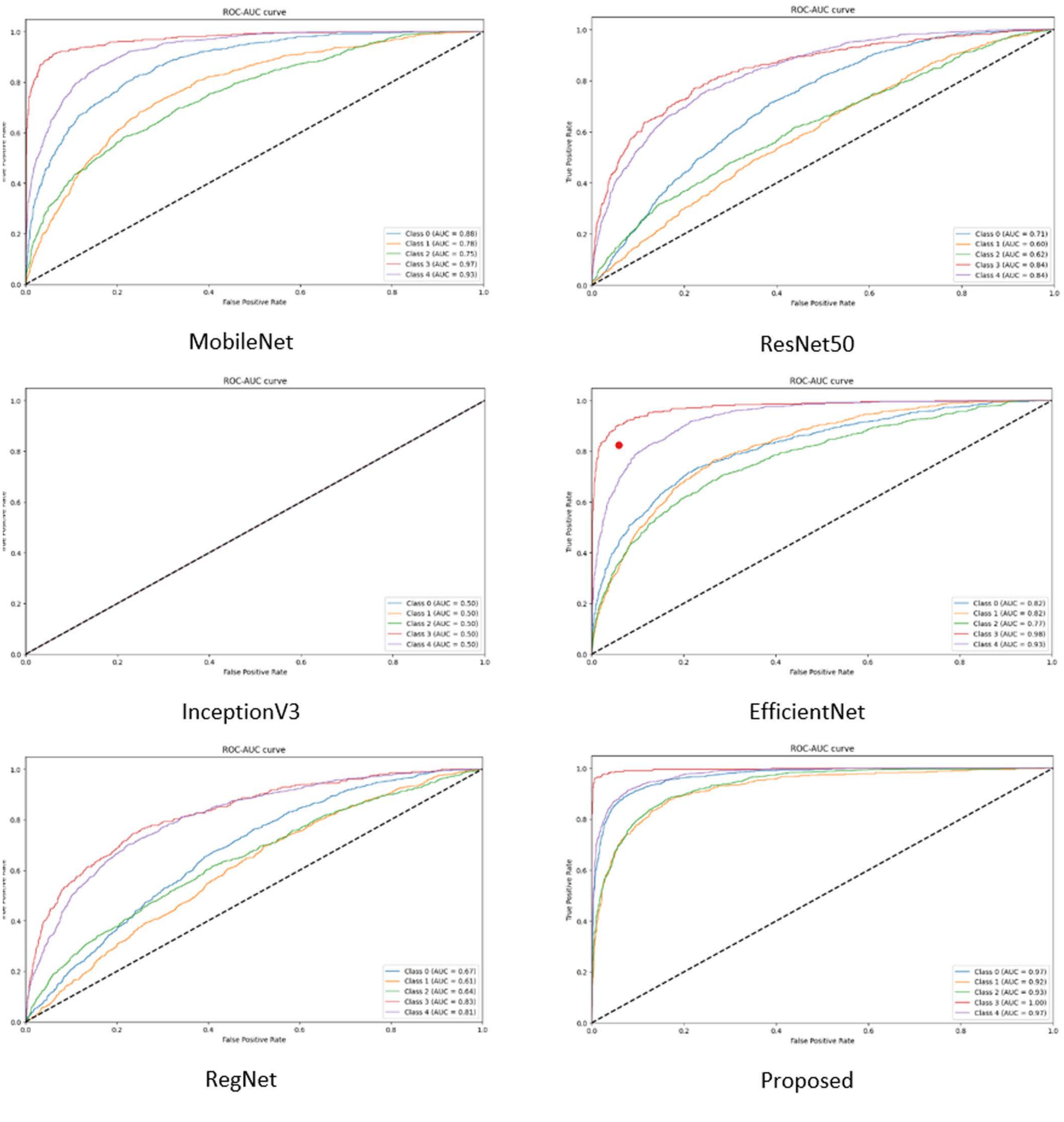

Fig 31. ROC-AUC curve for comparison.

https://doi.org/10.1371/journal.pone.0318530.g031

model proposed in this paper is comparable to that of the latest neural network structure, EfficientNet, and only inferior to the extremely lightweight MobileNet network. This proves that the proposed model can maintain faster processing speed and higher computational efficiency while outperforming similar models in terms of performance (e.g., accuracy, loss, etc.).

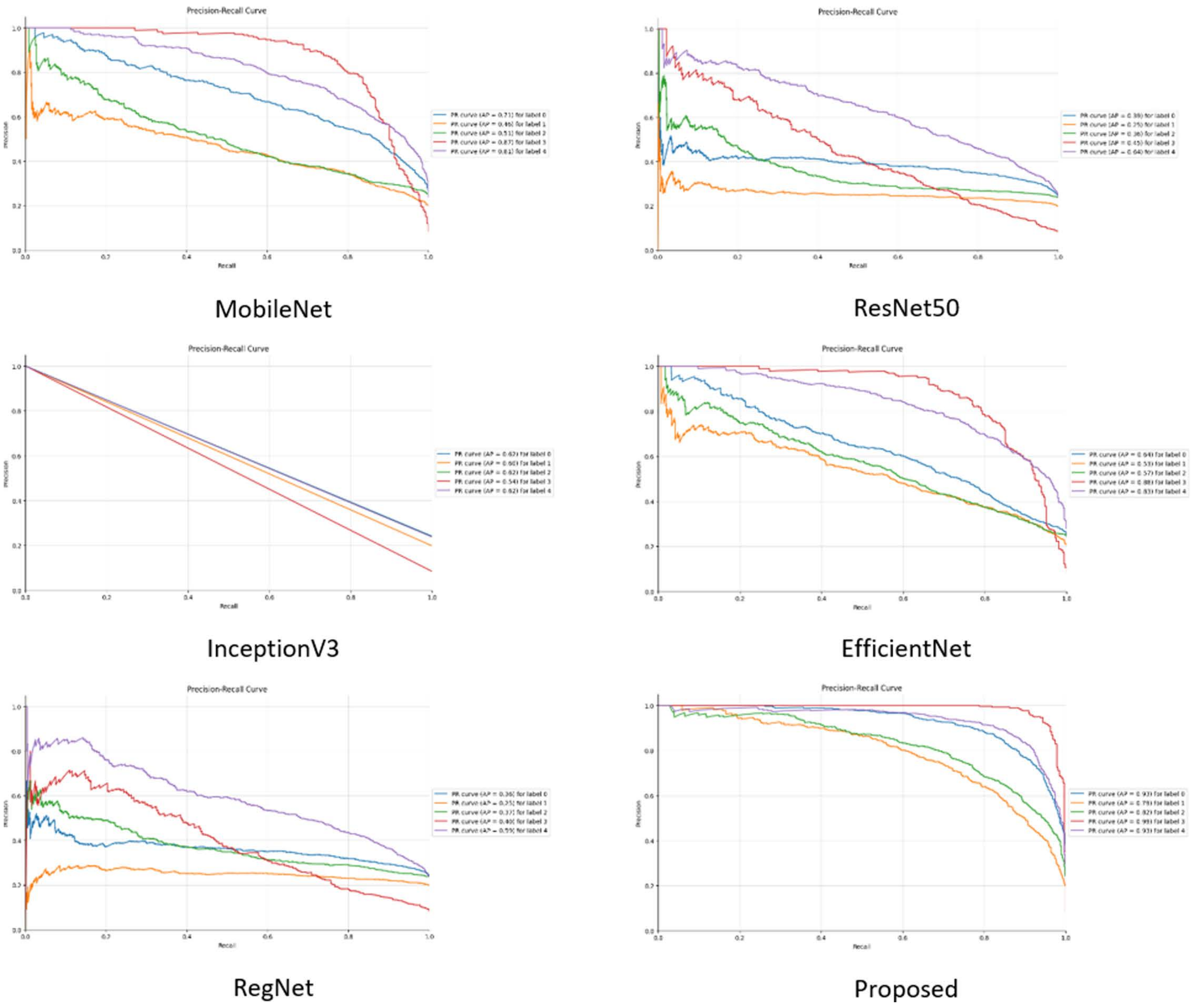

Fig 32. Precision-Recall curve for comparison.

https://doi.org/10.1371/journal.pone.0318530.g032

From the comparison of various metrics and the computational efficiency of the models on the test set for each of the above models, it can be seen that the embedding of the wavelet transform module in the neural network structure does not have an obvious impact on the computational speed of the models. Meanwhile, it can be seen from the confusion matrix and graphs that the proposed model can show high accuracy on each category, which is because the different dimensions of the image obtained after the introduction of the multilevel wavelet transform can be more easily focused on the main features of the image by the attention module in the model.

Indirect comparison with existing literature. As we can see in Table 16. Hedeya et al. [29] proposed a super-learner ensemble model that combines ResNet50, Xception, and

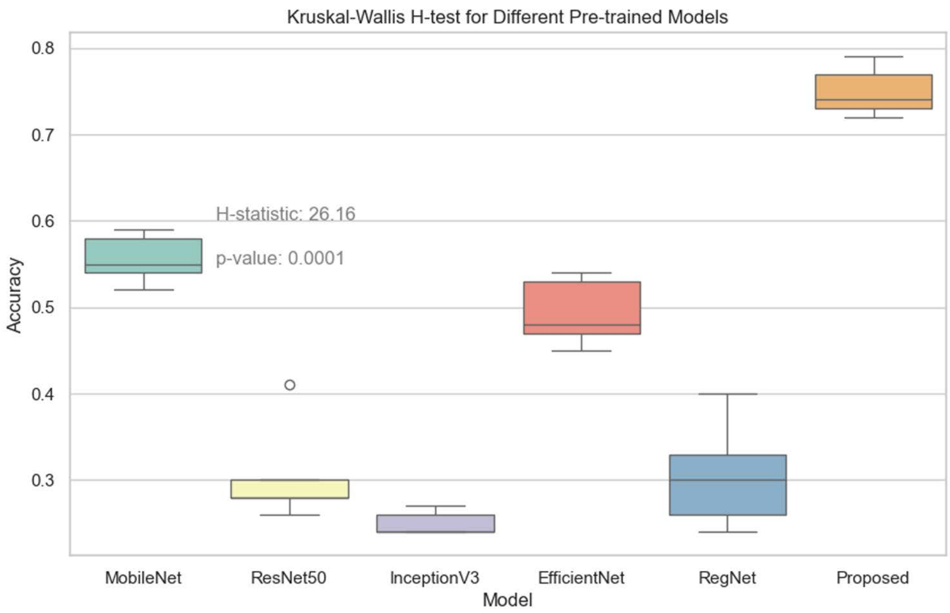

Fig 33. Kruskal-Wallis H-test for different pre-trained models.

https://doi.org/10.1371/journal.pone.0318530.g033

Table 15. Computational efficiency results of comparison with other existing models.

Model	Process time (number of samples// batch size)
MobileNet	0.0023
ResNet50	0.0052
InceptionV3	0.0084
EfficientNet	0.0043
RegNet	0.0288
**Proposed**	**0.0044**

https://doi.org/10.1371/journal.pone.0318530.t015

DenseNet to improve vehicle-type classification accuracy in traffic surveillance frames. This approach optimizes the strengths of individual models and achieves an overall accuracy of up to 97.94% on the MIO-TCD dataset and 97.62% on the BIT-vehicle dataset, demonstrating its effectiveness and robustness without relying on handcrafted features.

Soon et al. [30] proposed a semisupervised Principal Component Analysis Convolutional Network (PCN) that reduces training time and computational cost while maintaining high

Table 16. Indirect comparison with other methods.

Model	Vehicle Type	Accuracy (%)	Loss	Precision	Recall	F1	Size
Hedeya et al. [29]	Bus, Microbus, Minivan, Sedan, SUV, Truck	97.94	×	×	×	×	×
Soon et al. [30]	Microbus, Bus, Minivan, SUV, Sedan, Truck	88.35	×	×	×	×	×
Lin and Jhang [31]	Bus, Truck, Car, Motorcycle, Trailer	90.45	×	×	×	0.99	×
Kolukisa et al. [32]	Motorcycles, Passenger cars, Buses	91.15	×	×	×	0.915	×
Tan et al. [33]	SUV, Sedan, Microbus, Minivan, Truck, Bus	90.22	×	×	×	×	×
**DWAN**	**MPV, sedan, hatchback, pickup and sports**	**88.21**	**0.57**	**0.88**	**0.87**	**0.87**	**7.88M**

https://doi.org/10.1371/journal.pone.0318530.t016

accuracy in vehicle type classification. This is achieved by using PCA to generate convolutional filters, eliminating the need for time-consuming backpropagation training. The method is tested on the BIT-Vehicle dataset and achieves an average accuracy of above 88.35% using SoftMax and Support Vector Machine (SVM) classifiers, even under various challenging imaging conditions.

In the work of Lin and Jhang [31], a sophisticated system that combines the capabilities of YOLO for vehicle detection with Convolutional Fuzzy Neural Networks (CFNN) was introduced for vehicle classification and traffic flow counting. The system aims to monitor real-time traffic volume and vehicle types efficiently. Their system achieved an accuracy of 90.45% on the Beijing Institute of Technology public dataset and excelled with a mean average precision and F-measure (F1) of 99.00% on the GRAM-RTM dataset.

In addition, Kolukisa et al. [32] developed an intelligent system for vehicle-type classification using 3-D magnetic sensors and a deep neural network (DNN) approach with hyper-parameter optimization. Their system efficiently classified vehicles into light, medium, and heavy categories, achieving an accuracy of 91.15% and an f-measure of 91.50%.

The study by Tan et al. [33] presented a Spatial Attention Module (SAM) that enhances the high-level features derived from convolutional operations to improve classification accuracy in vehicle-type recognition tasks. The research aims to contribute to the field of vehicle recognition by providing a novel approach to improving classification accuracy, which has various practical applications in traffic monitoring, toll collection, and security enforcement. Their model achieved outstanding accuracy, notably reaching 84.48% and 95.96% accuracy on Stanford Cars and CompCarsWeb. Additionally, the model showed promise for real-time classification tasks, with inference times of 1 ms and 10 ms for CaffeNet-SAM and ResNet-SAM, respectively.

It is evident from the studies of the aforementioned researchers that while significant advancements have been made in vehicle classification methods, there remain areas for improvement. For instance, some models focus on specific vehicle types or are challenged by certain imaging conditions [30], while others may require extensive hyperparameter tuning and feature selection processes [32]. Despite these limitations, the collective efforts have pushed the boundaries in enhancing vehicle classification accuracy and efficiency in intelligent transportation systems.

Discussion

When comparing the DWAN, Res-DWAN, and Inception-DWAN models, the models show different strengths and limitations. The DWAN model, despite its simple structure, performs low in performance metrics such as accuracy, precision, recall, and F1-score, and the high loss values indicate that it may suffer from overfitting problems, which limits generalization capabilities. These problems can be ameliorated by introducing regularization techniques or optimizing the model structure. Res-DWAN shows excellent performance, especially in terms of high accuracy and low loss, but higher computational cost and model complexity may lead to increased resource usage and model maintenance costs. Therefore, techniques such as model pruning can be considered to simplify the model and maintain performance while reducing complexity. Inception-DWAN, on the other hand, while balanced in terms of overall performance, still has room for improvement in terms of category-specific recognition and the balance between precision and recall. In subsequent work, the feature extraction layer of the network can be adjusted or the category weights can be redesigned to improve the precision of category-specific recognition. Continuous iteration and optimization of the three models is the key to their success in real-world applications, and the utility and effectiveness of the models can be significantly improved through in-depth analysis and implementation of targeted optimization measures.

Conclusions

In conclusion, this study implements the classification of vehicles into selected categories including MPV, sedan, hatchback, pickup, and sports, based on the largest available dataset, CompCars. In this paper, after proposing and implementing the Depth-Wise Wavelet Attention Network (DWAN), two variants, Res-DWAN and Inception-DWAN, are also designed and experimentally demonstrated on the dataset with different aspects of the variants. In addition, this study realizes the domain-adaptive migration from web image data to traffic surveillance image data in the experimental stage and proves the effectiveness and good performance of the model on the datasets of two different scenarios. In the future, based on the model proposed in this paper and its variants, attempts can be made to apply the model to vehicle classification tasks in more scenarios, such as label classification and type classification. Given the idea of incorporating multilevel discrete wavelet transform and mixed-domain attention mechanism into deep network architecture in this paper, combining the wavelet transform with the attention mechanism will be considered to propose a novel attention module that retains the different advantages of attention mechanism and wavelet transform. In the new attention mechanism, the image is first abstracted into different levels by wavelet transform and then the important features of the image at each level will be captured by the self-attention mechanism. In this case, the capture of image features will be more refined and the interpretability of the model will be improved.

Acknowledgments

The authors would like to thank the editor and the anonymous reviewers for their valuable comments. This work was supported by Chengdu University of Technology.

Author contributions

Conceptualization: Rui Liu.

Data curation: Rui Liu, Shiyuan Wen.

Formal analysis: Rui Liu, Shiyuan Wen.

Investigation: Rui Liu.

Methodology: Rui Liu, Yufei Xing.

Project administration: Rui Liu, Shiyuan Wen.

Resources: Rui Liu.

Software: Rui Liu.

Supervision: Shiyuan Wen, Yufei Xing.

Validation: Yufei Xing.

Visualization: Shiyuan Wen.

Writing – original draft: Rui Liu, Shiyuan Wen, Yufei Xing.

Writing – review & editing: Shiyuan Wen.

References

1. Liu Z, Li Z, Li L, Yang H. Complex background classification network: A deep learning method for urban images classification. Comput Electri Eng. 2020;87:106771. https://doi.org/10.1016/j.compeleceng.2020.106771
2. Zhang J, Yang S, Bo C, Zhang Z. Vehicle logo detection based on deep convolutional networks. Comput Electri Eng. 2021;90:107004. https://doi.org/10.1016/j.compeleceng.2021.107004

3. Ganapathy S, Ajmera D. An intelligent video surveillance system for detecting the vehicles on road using refined YOLOV4. Comput Electric Eng. 2024;113:109036. https://doi.org/10.1016/j.compeleceng.2023.109036
4. Mou L, Ghamisi P, Zhu XX. Deep recurrent neural networks for hyperspectral image classification. IEEE Trans Geosci Remote Sensing. 2017;55(7):3639–55. https://doi.org/10.1109/tgrs.2016.2636241
5. Vaswani A, Shazeer N, Parmar N, Uszkoreit J, Jones L, Gomez AN, et al. Attention is all you need. Comput Sci Comput Lang. 2023 [Online]. https://doi.org/10.48550/arXiv.1706.03762
6. Kang E, Chang W, Yoo J, Ye JC. Deep convolutional framelet denosing for low-dose CT via wavelet residual network. IEEE Trans Med Imaging. 2018;37(6):1358–69. https://doi.org/10.1109/tmi.2018.2823756
7. Ma H, Liu D, Xiong R, Wu F. iWave: CNN-based wavelet-like transform for image compression. IEEE Trans Multimedia. 2020;22(7):1667–79. https://doi.org/10.1109/tmm.2019.2957990
8. Dong C, Loy CC, He K, Tang X. Image super-resolution using deep convolutional networks. IEEE Trans Pattern Anal Mach Intell. 2016;38(2):295–307. https://doi.org/10.1109/TPAMI.2015.2439281 PMID: 26761735
9. Eldele E, Chen Z, Liu C, Wu M, Kwoh C-K, Li X, et al. An attention-based deep learning approach for sleep stage classification with single-channel EEG. IEEE Trans Neural Syst Rehabil Eng. 2021;29:809–18. https://doi.org/10.1109/TNSRE.2021.3076234 PMID: 33909566
10. Simonyan K, Zisserman A. Very deep convolutional networks for large-scale image recognition. 2014. [Online]. Available from: https://arxiv.org/abs/1409.1556. https://arxiv.org/abs/1409.1556
11. Krizhevsky A, Sutskever I, Hinton GE. ImageNet classification with deep convolutional neural networks. Commun ACM. 2017;60(6):84–90. https://doi.org/10.1145/3065386
12. He K, Zhang X, Ren S, Sun J. Deep residual learning for image recognition. 2016 IEEE Conference on Computer Vision and Pattern Recognition (CVPR). 2016. p. 770–8. https://doi.org/10.1109/cvpr.2016.90
13. Daubechies I. The wavelet transform, time-frequency localization and signal analysis. IEEE Trans Inform Theory. 1990;36(5):961–1005. https://doi.org/10.1109/18.57199
14. Chen Y, Zhu W, Yao D, Zhang L. Vehicle type classification based on convolutional neural network. 2017 Chinese Automation Congress (CAC). 2017. p. 1898–901. https://doi.org/10.1109/cac.2017.8243078
15. Zhao D, Chen Y, Lv L. Deep reinforcement learning with visual attention for vehicle classification. IEEE Trans Cogn Dev Syst. 2017;9(4):356–67. https://doi.org/10.1109/tcds.2016.2614675
16. Nasaruddin N, Muchtar K, Afdhal A. A lightweight moving vehicle classification system through attention-based method and deep learning. IEEE Access. 2019;7:157564–73. https://doi.org/10.1109/access.2019.2950162
17. Awang S, Azmi NMAN, Rahman MdA. Vehicle type classification using an enhanced sparse-filtered convolutional neural network with layer-skipping strategy. IEEE Access. 2020;8:14265–77. https://doi.org/10.1109/access.2019.2963486
18. Guo L, Li R, Jiang B. An ensemble broad learning scheme for semisupervised vehicle type classification. IEEE Trans Neural Netw Learn Syst. 2021;32(12):5287–97. https://doi.org/10.1109/TNNLS.2021.3083508 PMID: 34086583
19. Zhao J, Hao S, Dai C, Zhang H, Zhao L, Ji Z, et al. Improved vision-based vehicle detection and classification by optimized YOLOv4. IEEE Access. 2022;10:8590–603. https://doi.org/10.1109/access.2022.3143365
20. Yu Y, Liu H, Fu Y, Jia W, Yu J, Yan Z. Embedding pose information for multiview vehicle model recognition. IEEE Trans Circuits Syst Video Technol. 2022;32(8):5467–80. https://doi.org/10.1109/tcsvt.2022.3151116
21. Park HL, Byun SH, Lee HS. Application of deep learning method for real-time traffic analysis using UAV. J Korean Soc Survey Geodesy Photogram Cartogr. 2020;38(4):353–61. https://doi.org/10.7848/ksgpc.2020.38.4.353
22. Xu J, Zhao J, Liu C. An effective hyperspectral image classification approach based on discrete wavelet transform and dense CNN. IEEE Geosci Remote Sensing Lett. 2022;19:1–5. https://doi.org/10.1109/lgrs.2022.3181627
23. Duan L, Xu D, Tsang IW-H. Domain adaptation from multiple sources: a domain-dependent regularization approach. IEEE Trans Neural Netw Learn Syst. 2012;23(3):504–18. https://doi.org/10.1109/TNNLS.2011.2178556 PMID: 24808555
24. Jaderberg M, Simonyan K, Zisserman A, Kavukcuoglu K. Spatial transformer networks. 2016. [Online]. Available from: https://arxiv.org/abs/1506.02025.

25. Hu J, Shen L, Albanie S, Sun G, Wu E. Squeeze-and-excitation networks. 2019. [Online]. Available: https://arxiv.org/abs/1709.01507.

26. Fu J, Liu J, Tian H, Li Y, Bao Y, Fang Z, et al. Dual attention network for scene segmentation. 2019 IEEE/CVF Conference on Computer Vision and Pattern Recognition (CVPR). 2019. p. 3141–9. https://doi.org/10.1109/cvpr.2019.00326

27. Woo S, Park J, Lee JY, Kweon IS. CBAM: convolutional block attention module. In: Computer Vision – ECCV 2018. Cham: Springer International Publishing; 2018. p. 3–19. https://link.springer.com/chapter/10.1007/978-3-030-01234-2_1

28. Guo M-H, Xu T-X, Liu J-J, Liu Z-N, Jiang P-T, Mu T-J, et al. Attention mechanisms in computer vision: A survey. Comp Visual Media. 2022;8(3):331–68. https://doi.org/10.1007/s41095-022-0271-y

29. Hedeya MA, Eid AH, Abdel-Kader RF. A super-learner ensemble of deep networks for vehicle-type classification. IEEE Access. 2020;8:98266–80. https://doi.org/10.1109/access.2020.2997286

30. Soon FC, Khaw HY, Chuah JH, Kanesan J. Semisupervised PCA convolutional network for vehicle type classification. IEEE Trans Veh Technol. 2020;69(8):8267–77. https://doi.org/10.1109/tvt.2020.3000306

31. Lin C-J, Jhang J-Y. Intelligent traffic-monitoring system based on yolo and convolutional fuzzy neural networks. IEEE Access. 2022;10:14120–33. https://doi.org/10.1109/access.2022.3147866

32. Kolukisa B, Yildirim VC, Ayyildiz C, Gungor VC. A deep neural network approach with hyper-parameter optimization for vehicle type classification using 3-D magnetic sensor. Comput Stand Interf. 2023;84:103703. https://doi.org/10.1016/j.csi.2022.103703

33. Tan SH, Chuah JH, Chow C-O, Kanesan J. Spatially recalibrated convolutional neural network for vehicle type recognition. IEEE Access. 2023;11:142525–37. https://doi.org/10.1109/access.2023.3342109
